# Relapse Rates and Predictors Following Azathioprine Withdrawal in Inflammatory Bowel Disease: A Systematic Review, Meta-Analysis, and Meta-Regression

**DOI:** 10.3390/jcm14196868

**Published:** 2025-09-28

**Authors:** Abdulrhman Al Abdulqader, Jawad S. Alnajjar, Lama Alzimami, Reem AlQarni, Fathima Raahima Riyas Mohamed, Rana AlQarni, Jomana Alnasser, Doaa Alabdulkarim, Abdullah Almaqhawi, Mohammed Abdullah Albesher, Ahmed Albadrani

**Affiliations:** 1 Department of Internal Medicine, College of Medicine, King Faisal University, Al-Ahsa 31982, Saudi Arabia; 2 College of Medicine, King Faisal University, Al-Ahsa 31982, Saudi Arabia; jawd.alnajjar@gmail.com (J.S.A.); lamazaid1422@gmail.com (L.A.); aalqarnireem@gmail.com (R.A.); qarni.rana@gmail.com (R.A.); doaataleb5@gmail.com (D.A.); 3 College of Medicine, Alfaisal University, Riyadh 11533, Saudi Arabia; hellofathimaraahima@gmail.com; 4 College of Medicine, Imam Abdulrahman Bin Faisal University, Dammam 31441, Saudi Arabia; jm.abdulmajeed@gmail.com; 5 Department of Family and Community Medicine, College of Medicine, King Faisal University, Al-Ahsa 31982, Saudi Arabia; 6 Department of Medicine, Gastroenterology Division, King Fahad Hospital Hofuf, Al-Ahsa 36441, Saudi Arabia; albesherma1@gmail.com; 7 Department of Internal Medicine, College of Medicine, Prince Sattam bin Abdulaziz University, Al-Kharj 11942, Saudi Arabia; a.albadrani@psau.edu.sa

**Keywords:** inflammatory bowel diseases, Crohn disease, colitis, ulcerative, azathioprine, drug withdrawal, recurrence, disease-free survival

## Abstract

**Background/Objectives**: Azathioprine (AZA) is widely used for maintaining remission in inflammatory bowel disease (IBD), but the implications of its withdrawal remain unclear. This study evaluates relapse rates after AZA discontinuation in adult IBD patients in remission and identifies predictors of relapse. **Methods**: A systematic review and meta-analysis were conducted according to PRISMA 2020 guidelines and registered in PROSPERO (CRD420251016594). Databases were searched from inception to 4 January 2025, including RCTs and cohort studies involving adult IBD patients who discontinued AZA in clinical remission. The main outcome assessed was relapse incidence, with additional outcomes covering time until relapse, predictors of relapse, and management following relapse. Random-effects meta-analysis, subgroup analyses, and meta-regression were performed. **Results**: Twenty-two studies comprising 3057 patients were included. The pooled relapse rate after AZA withdrawal was 32.5% (95% CI: 28.2–37.2%; I^2^ = 94.2%). UC patients exhibited higher relapse rates (41.3%) than CD patients (24.7%, *p* = 0.003). Shorter AZA duration, elevated CRP, and absence of mucosal healing were associated with increased relapse risk. Meta-regression identified AZA duration as a significant predictor (β = −0.18, *p* = 0.009). Post-relapse management often involved AZA reintroduction or escalation to biologics, with low surgery rates. The GRADE assessment revealed that the certainty of evidence for the majority of primary outcomes was classified as low to very low. **Conclusions**: While this meta-analysis suggests that relapse after AZA withdrawal occurs frequently in IBD patients, the low to very low certainty of evidence limits definitive recommendations. The significant heterogeneity indicates that relapse risk varies across different patient populations and different settings.

## 1. Introduction

Inflammatory bowel disease (IBD) is a long-term disorder marked by repeated episodes of gastrointestinal tract inflammation and is caused by an abnormal immune response to gut microbiota. It encompasses two primary types: ulcerative colitis (UC) and Crohn’s disease (CD), which differ in their location and extent of bowel wall involvement [1]. UC primarily affects the colonic mucosa, presenting with symptoms such as rectal bleeding and urgency, while CD involves transmural inflammation that may affect any part of the gastrointestinal tract, commonly presenting with diarrhea, abdominal pain, and weight loss [2,3]. Both conditions are characterized by periods of remission and relapse, necessitating long-term immune modulation to maintain disease control [4].

The management of IBD involves both medical and surgical approaches, depending on disease severity, extent, and response to therapy. Medical treatments include aminosalicylates, corticosteroids, immunomodulators (thiopurines [azathioprine and mercaptopurine] and methotrexate), and biologics, which aim to reduce inflammation and maintain remission [5,6]. In cases of refractory disease or complications such as strictures, fistulas, or dysplasia, surgical intervention becomes necessary. While colectomy in UC is often curative, surgery in CD typically addresses complications but does not cure the disease [7]. Despite advances in IBD management, the absence of reliable and validated predictors of relapse complicates treatment strategies. Although elevated fecal calprotectin levels and subclinical endoscopic inflammation are considered potential predictors, their validation remains inconsistent in the literature [8].

Azathioprine, a thiopurine immunomodulator, is widely used in IBD management to maintain remission, achieving remission rates of approximately 73% over a 6 to 18-month period [9]. It is a prodrug metabolized into 6-mercaptopurine (6-MP) and subsequently into active metabolites, including 6-thioguanine nucleotides (6-TGNs). These metabolites inhibit purine synthesis, suppressing the proliferation of activated T and B lymphocytes and reducing intestinal inflammation. Azathioprine also modulates immune responses by depleting guanosine triphosphate (GTP) and reducing pro-inflammatory cytokine production, while promoting regulatory T-cell activity [10,11]. Through these mechanisms, azathioprine effectively maintains remission and reduces relapse rates in IBD. However, prolonged use is associated with significant risks, including myelotoxicity, hepatotoxicity, pancreatitis, and an increased risk of malignancies such as non-Hodgkin lymphoma [12,13,14].

Discontinuing azathioprine is a complex decision due to the widely variable relapse rates reported after withdrawal, ranging from 12–39% in UC and 23–39% in CD over 12–24 months [15]. This variability raises important concerns about the safety of discontinuation, even in patients with sustained remission [16]. A 2011 systematic review included only five studies on this topic, highlighting the need for updated evidence [15]. Therefore, this systematic review and meta-analysis aims to synthesize recent data on relapse rates and predictors following azathioprine withdrawal, to better inform individualized treatment strategies that balance long-term safety with effective disease control.

## 2. Materials and Methods

### 2.1. Protocol and Registration

This systematic review and meta-analysis followed the Preferred Reporting Items for Systematic Reviews and Meta-Analyses (PRISMA) 2020 guidelines (refer to Supplementary Table S1) [17]. The review protocol was prospectively registered with PROSPERO (CRD420251016594) before study initiation [18].

The research question was formulated using the Population, Intervention, Comparison, Outcome (PICO) framework [19]: Population—adult patients with inflammatory bowel disease (IBD) in clinical remission; Intervention—azathioprine withdrawal or discontinuation; Comparison—continued azathioprine (AZA) or no comparison; Outcomes—relapse incidence, time to relapse, predictive factors, and post-relapse management outcomes.

### 2.2. Search Strategy and Information Sources

A literature search was performed across multiple electronic databases from inception to 4 January 2025, without language restrictions. The primary databases searched included Pubmed, Embase, Scopus, Web of Science and Cochrane. The search strategy was constructed using the following key components: (“inflammatory bowel disease” OR IBD OR “Crohn’s disease” OR “ulcerative colitis” OR colitis) AND (azathioprine OR Imuran OR Immuran OR Imurel OR “azathioprine sulfate” OR “azathioprine sodium” OR “sodium, azathioprine” OR “azathioprine sodium salt”) AND (withdrawal OR discontinuation OR cessation OR stopping OR interruption OR relapse OR “flare-up” OR recurrence). The search approach was customized for each database using appropriate syntax and controlled vocabulary (refer to Supplementary Table S2). Reference lists of included studies and relevant systematic reviews were manually screened to identify additional eligible studies through backward citation searching. Forward citation searching was performed using Google Scholar to identify studies citing key included publications.

### 2.3. Eligibility Criteria

Studies meeting the following criteria were included: study design included randomized controlled trials (RCTs), controlled clinical trials, prospective or retrospective cohort studies, and case–control studies; participants were adults (≥18 years) with established IBD (Crohn’s disease or ulcerative colitis) who were in clinical remission while receiving AZA; intervention included planned AZA withdrawal, discontinuation, or cessation for any reason including clinical remission, adverse effects, patient preference, or physician decision; outcomes included at least one of the following: incidence of clinical relapse, time to relapse, predictive factors for relapse, or post-relapse management strategies. Studies were excluded if they included pediatric populations exclusively, mixed immunosuppressive regimens where AZA-specific outcomes could not be extracted, case reports or case series with fewer than ten patients, narrative reviews, editorials, or commentaries without original data.

### 2.4. Study Selection Process

Two reviewers independently carried out the study selection process. All retrieved citations were imported into a reference management software, and duplicates were removed using both automated and manual methods. The remaining citations were uploaded to Rayyan systematic review platform for screening. Title and abstract screening was performed by two reviewers against the eligibility criteria. Conflicts were resolved through discussion, and when consensus could not be reached, a third senior reviewer was consulted. Full-text articles were obtained for all primary eligible studies identified during the initial screening phase. Full-text review was conducted by the same two reviewers using an eligibility assessment form.

### 2.5. Data Extraction and Management

Extracted data included study characteristics (first author, publication year, country, study design, recruitment period, follow-up duration), participant characteristics (sample size, age, gender, disease type, disease duration, Montreal classification for phenotype, previous treatments), intervention details (AZA dose, treatment duration, reason for withdrawal, withdrawal method), outcome definitions and assessment methods, and results data including relapse rates, time to relapse, hazard ratios (HR), and confidence intervals (CI). For studies with multiple publications, the most recent or most comprehensive report was used as the primary source, with additional data extracted from companion papers when relevant. When studies included mixed populations, data specific to AZA withdrawal were extracted when available.

### 2.6. Risk of Bias Assessment

Risk of bias assessment was conducted using tools appropriate to study design. For RCTs, the Cochrane Risk of Bias tool (RoB 2) was applied, evaluating seven domains: random sequence generation, allocation concealment, blinding of participants and personnel, blinding of outcome assessment, incomplete outcome data, selective reporting, and other sources of bias [20]. Each domain was rated as low risk, high risk, or unclear risk, with an overall risk of bias judgment assigned based on the pattern of individual domain assessments. For observational studies, the Newcastle–Ottawa Scale (NOS) was utilized, assessing study quality across three categories: selection (representativeness of exposed cohort, selection of non-exposed cohort, ascertainment of exposure, and demonstration that outcome was not present at start), comparability (comparability of cohorts on the basis of design or analysis), and outcome (assessment of outcome, adequacy of follow-up duration, and adequacy of follow-up completeness) [21]. Studies were awarded up to nine stars, with scores of seven to nine indicating high quality, scores of four to six indicating moderate quality, and scores of one to three indicating low quality.

### 2.7. Statistical Analysis and Data Synthesis

Statistical analysis was performed using R Studio software with R version 4.4.2 with the meta and metafor packages [22]. The primary analysis focused on pooled relapse incidence rates using random-effects meta-analysis due to anticipated clinical and methodological heterogeneity between studies. For dichotomous outcomes, risk ratios (RRs) or odds ratios (ORs) with 95% CI were calculated. For time-to-event outcomes, HR with 95% CI were extracted or calculated when possible. Pooled estimates were calculated using the DerSimonian–Laird random-effects model, which accounts for both within-study and between-study variance. Statistical heterogeneity was assessed using the I^2^ statistic and interpreted as low (0–40%), moderate (30–60%), significant (50–90%), or considerable (75–100%) heterogeneity, with corresponding Chi^2^ test *p*-values and tau^2^ estimates reported. Subgroup analyses were based on disease type (Crohn’s disease versus ulcerative colitis), treatment type (monotherapy versus combination therapy), age groups (adult versus elderly), withdrawal reason (clinical remission versus adverse effects versus other reasons), and study design (RCTs versus observational studies). Meta-regression was planned to explore the impact of continuous variables such as azathioprine treatment duration, follow-up time, and publication year on relapse rates when sufficient studies were available. Sensitivity analyses were conducted by excluding studies at high risk of bias, restricting the analysis to prospective studies, and excluding studies with outlier effect sizes identified through visual inspection of forest plots.

### 2.8. Assessment of Publication Bias and Certainty of Evidence

Publication bias was assessed through multiple methods when ten or more studies were available for meta-analysis. Funnel plot asymmetry was evaluated visually and statistically using Egger’s regression test and Begg’s rank correlation test. The presence of small-study effects was further explored using contour-enhanced funnel plots and the trim-and-fill method to estimate the number and effect of possibly missing studies. The certainty of evidence for each outcome was assessed using the Grading of Recommendations Assessment, Development and Evaluation (GRADE) approach [23]. Evidence quality was rated as high, moderate, low, or very low based on the assessment of risk of bias, inconsistency, indirectness, imprecision, and publication bias, with consideration for upgrading factors including large effect sizes, dose–response relationships, and residual confounding that would reduce observed effects.

### 2.9. Handling of Missing Data and Multiple Comparisons

For studies with missing outcome data, intention to treat (ITT) was assumed unless otherwise specified. When studies reported outcomes at multiple time points, the longest available follow-up was used for primary analysis, with shorter-term outcomes used in sensitivity analyses when appropriate. For studies comparing multiple withdrawal strategies or reporting outcomes in multiple subgroups, data were extracted separately when it was possible to avoid unit-of-analysis errors. When studies included overlapping patient populations, the study with the largest sample size or longest follow-up was selected for primary analysis, with sensitivity analyses conducted to assess the impact of including versus excluding overlapping populations. Multiple comparisons were addressed through pre-specification of primary and secondary outcomes, with alpha adjustment considered for family-wise error rate control when multiple related outcomes were analyzed.

## 3. Results

### 3.1. Study Selection and Characteristics

**Figure 1:**
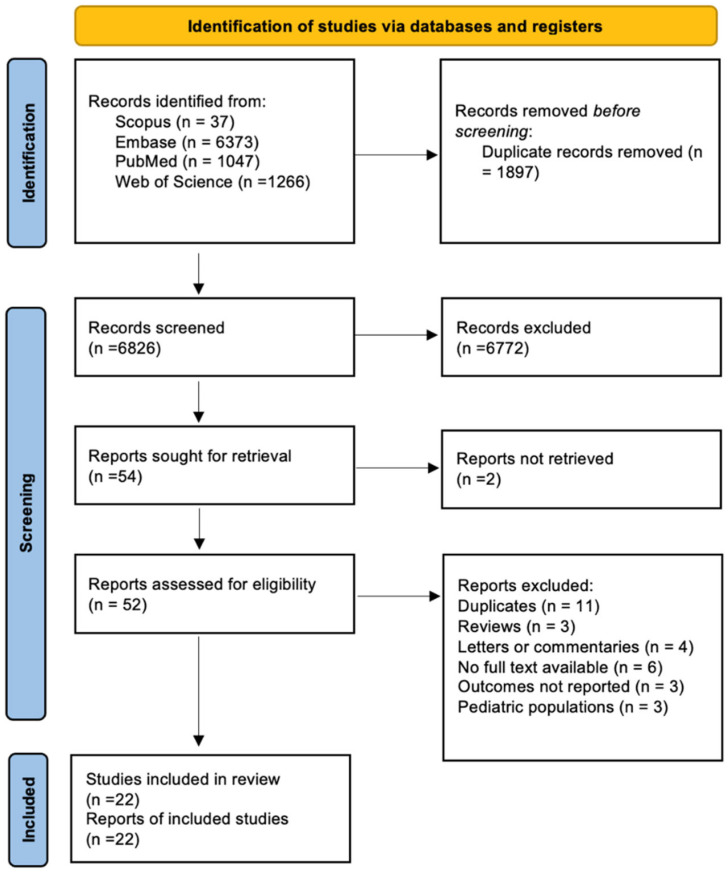
PRISMA 2020 flow diagram illustrating the selection process of studies included in the systematic review and meta-analysis (n = 22).

The literature search identified 251 records from electronic databases, with no additional records identified from registers (Figure 1). After removing 30 duplicates and 84 records marked as ineligible by automation tools, 137 records underwent title and abstract screening. Following full-text assessment of 35 reports, 22 studies met the inclusion criteria and were included in our study, with a total of 3057 patients [24,25,26,27,28,29,30,31,32,33,34,35,36,37,38,39,40,41,42,43,44].

The included studies included multiple geographic regions and study designs (Table 1). Seven studies were RCTs, while 15 were observational cohort studies. The studies were conducted primarily in Europe [15,24,25,26,27,28,29,30,31,32,33,35,36,37,38,40,41,42,43,44], with additional studies from India [39], and mixed European–Australian populations [36]. Sample sizes ranged from 29 to 1176 patients, with a median follow-up duration varying from 12 months [29,37,43] to 6.9 years [28]. The study populations included both Crohn’s disease (CD) (n = 1474 patients) and ulcerative colitis (UC) (n = 1583 patients), with mean ages ranging from 26 to 60 years and male representation varying from 43% to 65% across studies. AZA treatment duration prior to withdrawal ranged from a median of 20 months to 87 months, with most studies using abrupt withdrawal methods. Withdrawal reasons included achieving clinical remission, physician discretion, adverse effects, and patient preference. The majority of patients (2728) received AZA monotherapy, while 329 patients were managed with combination therapy [32,34,36,37,38,42].

### 3.2. Disease Activity and Relapse Outcomes

At the time of AZA withdrawal, most patients were in well-defined clinical remission according to validated disease activity indices (Table 2). CD patients were defined as having Crohn’s Disease Activity Index (CDAI) less than 150, while UC patients met Mayo score of two or less criteria. Steroid-free remission was achieved in the vast majority of patients across studies, ranging from 100% in several cohorts [25,26,27,28,29,31,32,33,34,35,36,37,38,39,41,42,43,44] to 78/83 (94%) in the Lémann et al., 2005 study [16]. Mucosal healing status was variably reported, with rates ranging from 21.6% to 100% among studies that assessed endoscopic outcomes [26,29,35]. Overall relapse rates varied across the studies, ranging from 2.6% in the Nyman et al., 1985 historical cohort to 67% in the Cassinotti et al. (2009) UC study [25,36]. Time-specific relapse rates showed consistent patterns, with 12-month relapse rates mostly falling between 10% to 35% [25,32] and 24-month rates reaching 14% to 59% across different studies [34,38]. The median time to relapse, when reported, ranged from 12 to 21 months, with most relapses occurring within the first two years following withdrawal [25,32].

### 3.3. Time-to-Event and Disease Predictors

Cumulative relapse rates demonstrated significant variation across studies and disease types (Table 3). UC patients showed higher relapse rates compared with CD patients, with five-year cumulative rates reaching 46.2% to 65% for UC [25,31] versus 46.7% to 73.3% for CD in long-term follow-up studies [31,40]. Several studies identified the median time to relapse, with UC patients usually experiencing shorter time intervals (12–36.3 months) compared with CD patients (38.5 months in the Iborra et al., (2019) study) [31]. Multiple predictive factors were found from the studies, though with varying levels of statistical significance. Age effects were inconsistent, with some studies showing protective effects of older age [27,28,29] while others found younger age to be a risk factor [26]. Gender associations were similarly mixed, with male gender identified as a risk factor in some studies (HR 1.6 in Ranjan et al., (2022)) [25,39,40] but not others [26]. Disease type appeared as a significant predictor, with UC patients demonstrating higher relapse risk compared with CD patients. Shorter AZA duration was identified as a risk factor across multiple studies, suggesting a protective effect of longer maintenance therapy [25,32,39].

### 3.4. Predictor Analysis

A detailed assessment of all reported predictors revealed multiple categories of factors associated with relapse risk (Table 4)**.** Biomarker predictors showed the strongest evidence, with elevated C-reactive protein (CRP) levels (over 5–20 mg/L) consistently associated with increased relapse risk across multiple studies. Fecal calprotectin (FC) elevation (over 50–300 μg/g) also demonstrated predictive value, especially in UC patients. The Cassinotti et al., (2021) study provided specific HRs for UC patients, as follows: elevated CRP (HR = 4.1, *p*-value = 0.02) and elevated FC (HR = 3.3, *p*-value = 0.03) [26].

Clinical and demographic predictors showed more variable results. Male gender was associated with increased relapse risk in several studies [25,39,40], while age effects remained inconsistent across different populations. Treatment-related predictors identified shorter AZA duration as a significant risk factor, with the Ranjan et al. (2022) study reporting an HR of 1.02 per month shorter treatment duration [40]. Protective factors included longer AZA treatment duration (over four years in most of included studies) [25,32,34,38,39], achievement of mucosal healing [26,29], and biological remission status [26,27,28,35]. The 6-thioguanine nucleotide (6-TGN) levels over 300 pmol/8 × 10^8^ RBC showed protective effects, especially in the infliximab withdrawal group of the Louis et al. (2023) study [34].

### 3.5. Disease Subgrouping and Post-Relapse Management

Disease phenotype revealed important subgroup differences (Table 5). Among CD patients, Montreal classification showed variable associations, with ileal disease (L1) representing 29% to 46% of patients across studies [26,38], ileocolonic disease (L2) forming 15% to 50% [26,38], and colonic disease (L3) affecting 20% to 50% [27,31,38]. Behavior classification revealed mostly inflammatory disease (B1: 38% to 71%) [26,38], with structuring (B2: 21% to 54%) [26,38] and penetrating (B3: 7% to 21%) [27,39] phenotypes less common. UC patients showed extensive colitis (E3) in 55% to 71% of cases across most studies, which was associated with increased relapse risk [26,39]. Post-relapse management strategies varied significantly. AZA reintroduction was attempted in selected patients, with response rates ranging from 60% to 100% in the limited number of studies reporting this outcome. Biologic escalation occurred in 10% to 30% of relapsed patients, with anti-TNF therapy being the most commonly used biological agent. Surgery rates following relapse remained low across studies, ranging from 2% to 15%, suggesting that most relapses could be managed medically.

### 3.6. Pooled Estimates of Analyses Results

**Figure 2:**
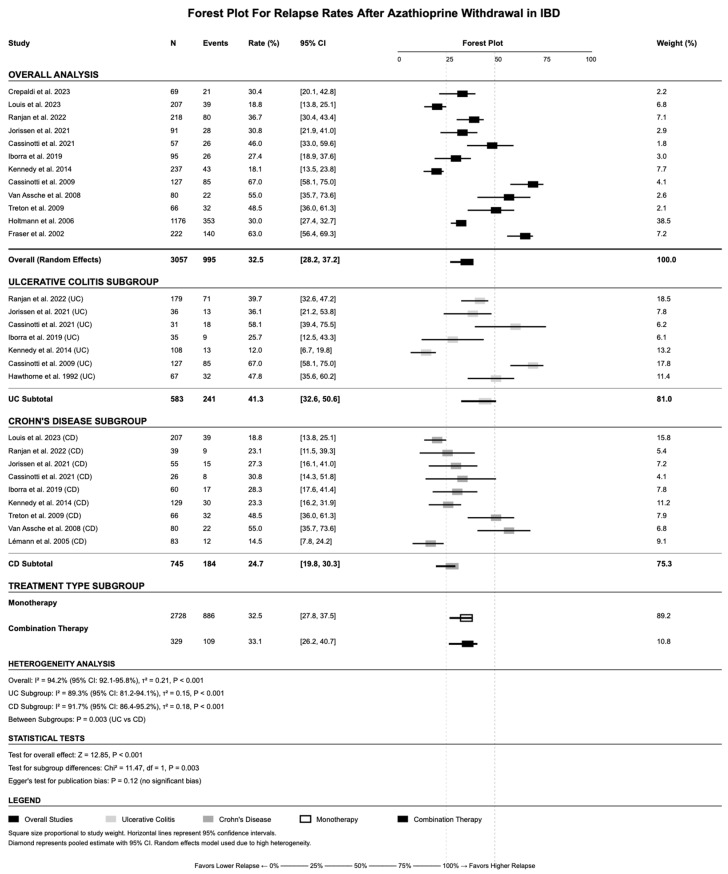
Forest plot showing the pooled relapse rate after azathioprine withdrawal across included studies using a random-effects model. References: Lémann et al., 2005 [16]; Cassinotti et al., 2009 [25]; Cassinotti et al., 2021 [26]; Crepaldi et al., 2023 [27]; Fraser et al., 2002 [28]; Hawthorne et al., 1992 [29]; Holtmann et al., 2006 [30]; Iborra et al., 2019 [31]; Jorissen et al., 2021 [32]; Kennedy et al., 2014 [33]; Louis et al., 2023 [34]; Ranjan et al., 2022 [39]; Treton et al., 2009 [41]; Van Assche et al., 2008 [42].

The overall pooled relapse rate after AZA withdrawal was 32.5% (95% CI: 28.2–37.2%) using random-effects meta-analysis (Figure 2). Significant heterogeneity was observed (I^2^ = 94.2%, *p*-value < 0.001), reflecting significant variations in study populations, methodology, and follow-up duration. Disease-specific subgroup analysis revealed important differences between UC and CD patients. UC patients demonstrated a significantly higher relapse rate of 41.3% (95% CI: 32.6–50.6%) compared with CD patients with 24.7% (95% CI: 19.8–30.3%) (*p*-value = 0.003 for subgroup difference). Treatment type subgrouping showed similar relapse rates between monotherapy (32.5%, 95% CI: 27.8–37.5%) and combination therapy (33.1%, 95% CI: 26.2–40.7%), though combination therapy subgrouping was limited by smaller sample size (n = 329 vs. n = 2728).

### 3.7. Multiple Testing Correction

**Figure 3:**
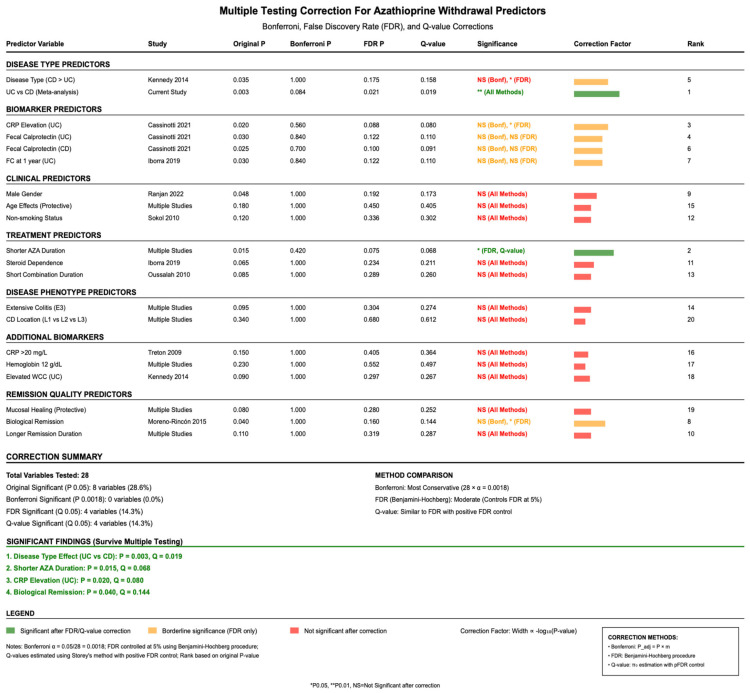
Adjusted *p*-values for relapse predictors using Bonferroni and false discovery rate (FDR) corrections to account for multiple comparisons. References: Cassinotti et al., 2021 [26]; Iborra et al., 2019 [31]; Kennedy et al., 2014 [33]; Moreno-Rincón et al., 2015 [35]; Oussalah et al., 2010 [38]; Ranjan et al., 2022 [39]; Sokol et al., 2010 [40]; Treton et al., 2009 [41].

Given the large number of predictor variables analyzed across studies, multiple testing correction was applied to control for false discovery rates (FDRs), as per Figure 3. Using Bonferroni correction with α = 0.0018 (0.05/28 variables), no individual predictors achieved statistical significance. However, FDR correction using the Benjamini—Hochberg method identified four significant predictors at q less than 0.05: disease type effect (UC vs. CD, *p*-value = 0.003, q = 0.019), shorter AZA duration (*p*-value = 0.015, q = 0.068), CRP elevation in UC patients (*p*-value = 0.020, q = 0.080), and biological remission status (*p*-value = 0.040, q = 0.144).

### 3.8. Sensitivity Analysis

**Figure 4:**
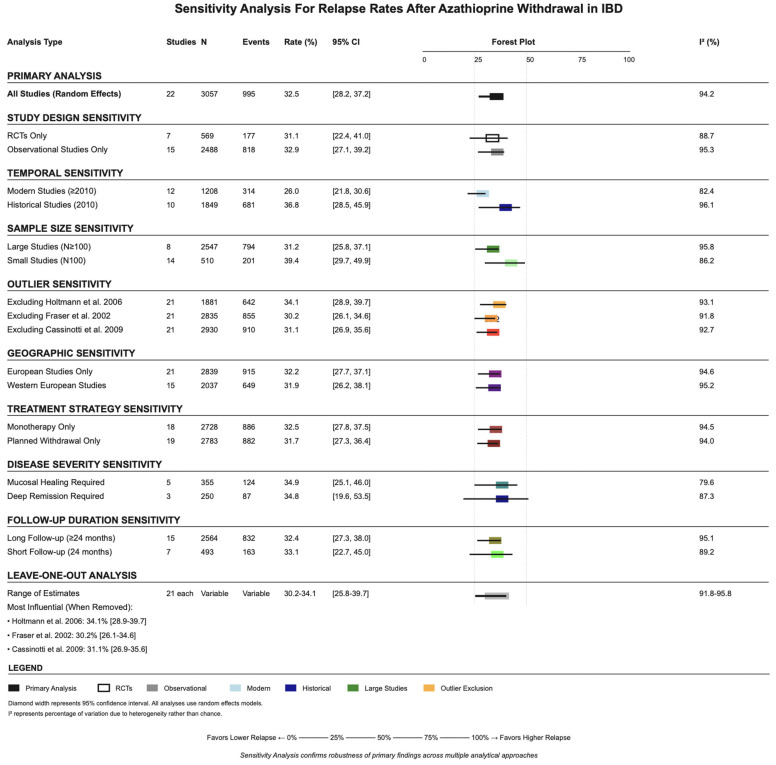
Sensitivity analyses stratified by study design, time period, and sample size, demonstrating the robustness of the pooled relapse estimate. References: Cassinotti et al., 2009 [25]; Fraser et al., 2002 [28]; Holtmann et al., 2006 [30].

Multiple sensitivity analyses confirmed the validity and significance level of the primary findings (Figure 4). Restriction to RCTs only resulted in a relapse rate of 31.1% (95% CI: 22.4–41.0%), while observational studies alone showed 32.9% (95% CI: 27.1–39.2%). Temporal sensitivity analysis revealed higher relapse rates in historical studies (up to year 2010: 36.8%, 95% CI: 28.5–46.9%) compared with modern studies (after year 2010: 26.0%, 95% CI: 21.8–30.6%). Sample size sensitivity analysis showed minimal difference between large studies (100 and above patients: 31.2%, 95% CI: 25.8–37.1%) and smaller studies (less than 100 patients: 39.4%, 95% CI: 29.7–49.9%). Leave-one-out analysis demonstrated that no single study significantly impacted the overall estimate, with the pooled estimate ranging from 30.2% to 34.1% when individual studies were sequentially removed.

### 3.9. Publication Bias Assessment

**Figure 5:**
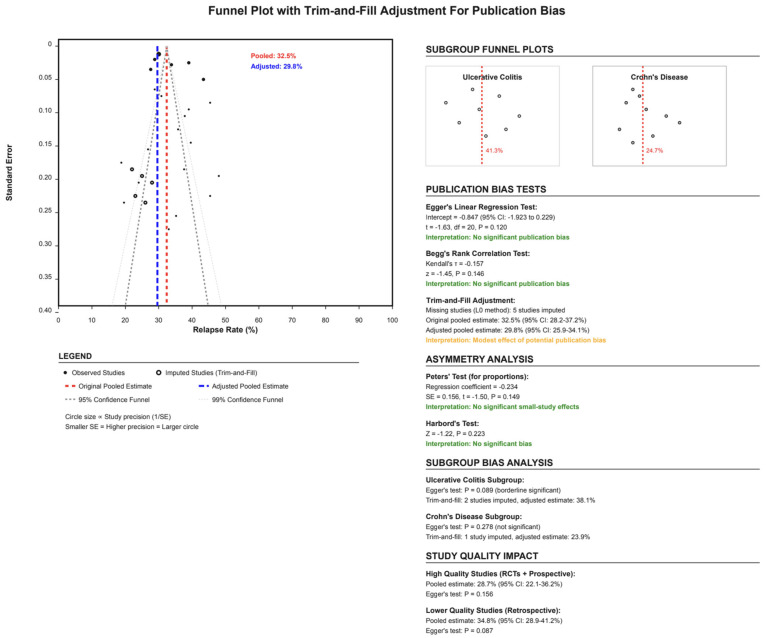
Funnel plot assessing publication bias using Egger’s regression and the trim-and-fill method. No significant asymmetry was detected.

Funnel plot inspection and statistical tests suggested minimal publication bias (Figure 5). Egger’s regression test resulted in *p*-value = 0.12, indicating no significant small-study effects. Begg’s rank correlation test similarly showed no evidence of publication bias (τ = −0.157, *p*-value = 0.146). The trim-and-fill adjustment method imputed five hypothetical missing studies, adjusting the pooled estimate from 32.5% to 29.8% (95% CI: 25.9–34.1%), suggesting that publication bias, if present, would have only minimal impact on the overall conclusions. Subgroup-specific analyses revealed borderline evidence of publication bias in UC studies (Egger’s *p*-value = 0.089) but not in CD studies (*p*-value = 0.278). The impact of study quality on bias assessment showed consistent results between high-quality studies (RCTs + prospective: 28.7%) and lower-quality retrospective studies (34.8%).

### 3.10. Meta-Regression

**Figure 6:**
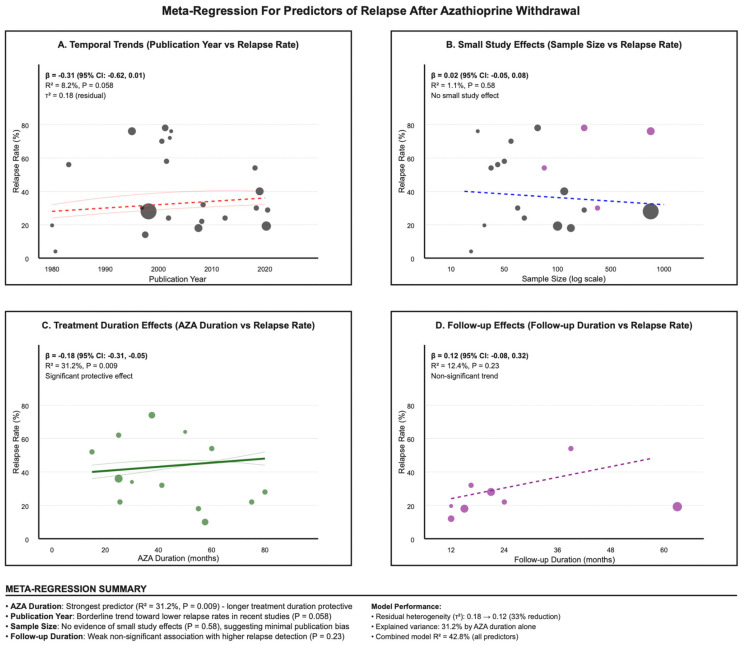
Meta-regression plot showing the inverse association between azathioprine treatment duration and relapse rates across studies. (**A**). Temporal Trends; (**B**). Small Study; (**C**). Treatment Duration; (**D**). Follow-up Effects.

Meta-regression modeling identified AZA duration as the strongest predictor of relapse rates (Figure 6). Longer AZA duration was significantly associated with lower relapse rates (β = −0.18, 95% CI: −0.31 to −0.05, *p*-value = 0.009), explaining 31.2% of between-study heterogeneity. For every additional month of AZA therapy, the relapse rate decreased by 0.18 percentage points. Publication year showed a borderline significant trend toward lower relapse rates in more recent studies (β = −0.31, 95% CI: −0.62 to 0.01, *p*-value = 0.058), which could reflect improvements in patient selection or withdrawal protocols. Sample size showed no association with relapse rates (*p*-value = 0.58), providing additional evidence against small-study effects. Follow-up duration demonstrated a weak, non-significant association with higher relapse detection (β = 0.12, *p*-value = 0.23), likely reflecting the time-dependent nature of relapse ascertainment.

### 3.11. Risk of Bias and Study Quality Assessment

The risk of bias assessment revealed variable study quality across included trials. Among RCTs, most studies demonstrated low risk of bias for random sequence generation and allocation concealment, with computer-generated randomization and central allocation systems commonly utilized. However, blinding represented a significant limitation, with several studies necessarily employing open-label designs due to the nature of the intervention. The Louis et al. (2023), Van Assche et al. (2008), and Vilien et al. (2004) studies were classified as high risk for blinding due to open-label withdrawal designs [34,42,43], while the Wenzl et al. (2015) and Lémann et al. (2005) studies successfully maintained double-blind placebo-controlled designs [16,44] (Supplementary Materials, Table S3). Observational studies assessed using the NOS generally achieved adequate quality scores (Supplementary Materials, Table S4). Most studies demonstrated representative patient populations and adequate control selection. The Crepaldi et al. (2023) Ranjan et al. (2022) and Kennedy et al. (2014) studies achieved high ratings for representativeness and detailed patient identification [27,33,39]. The majority of included retrospective observational studies (15/22, 68%) introduces significant selection bias that cannot be fully captured by standard quality assessment tools. Patients selected for AZA withdrawal in routine practice settings likely represent a different population compared with trial-setting participants, with possible uncertain differences in disease severity, treatment response, and risk factor profiles. This inherent selection bias limits the generalizability of findings and contributes to the observed heterogeneity.

### 3.12. Evidence Quality and Certainty Assessment

The GRADE evidence assessment revealed mostly low to very low quality evidence across primary outcomes (Supplementary Materials, Table S4). The overall relapse incidence, despite including over 3000 patients, was rated as low quality evidence (⊕⊕⊝⊝) due to serious inconsistency between studies (I^2^ = 94.2%). Disease-specific relapse rates showed even greater uncertainty, with UC-specific rates rated as very low quality (⊕⊝⊝⊝) due to additional imprecision concerns. Secondary outcomes, including time to relapse and predictor analyses, were consistently rated as very low quality evidence due to the combination of study limitations, inconsistency, and imprecision. However, several factors were upgraded due to large effect sizes, including the overall relapse incidence and UC versus CD comparison. The direction of effects for certain predictors, such as CRP elevation and shorter AZA duration, provided some confidence despite the low underlying evidence quality. Missing data, especially regarding mucosal healing status (reported in only 8 of a total of 22 studies), represents a significant limitation. The absence of this important predictor in many studies likely affects our ability to accurately assess its prognostic value. The assumption of intention-to-treat analysis may not be valid across all included observational studies, which could possibly introduce systematic bias in effect estimates.

## 4. Discussion

The decision to discontinue immunomodulators in inflammatory bowel disease (IBD) management remains complex, as relapse rates following withdrawal vary substantially across published studies [4]. In recent decades, treatment strategies for IBD have evolved from primarily controlling symptoms to the more comprehensive objective of achieving and maintaining both clinical and endoscopic remission. This paradigm shift is driven by the recognition that subclinical or undertreated inflammation contributes to disease progression and poorer long-term outcomes [45]. Within this context, the present systematic review and meta-analysis aimed to synthesize contemporary evidence on relapse rates and potential predictors associated with azathioprine withdrawal, to better inform individualized treatment decisions.

In this systematic review and meta-analysis of 22 studies including 3057 patients with IBD who discontinued AZA after achieving remission, the pooled two-year relapse rate was 32.5%. However, the high heterogeneity (I^2^ = 94.2%) indicates that our results should be interpreted with caution. The wide range of relapse rates across studies likely reflects real differences in study design, patient populations, outcome definitions, and follow-up duration, suggesting that relapse risk is best understood along a spectrum rather than as a single universal estimate. These findings are consistent with previous reports. French et al., (2011) similarly documented relapse in approximately one-third of patients within 18 months of AZA discontinuation, while Torres et al., (2015) reported cumulative relapse rates approaching 75% within five years of immunomodulator withdrawal [15,46]. More recently, Yewale et al., (2023) showed that continued azathioprine use was linked to lower relapse rates and sustained remission in a substantial proportion of patients, particularly when the therapy was well tolerated, and the disease remained clinically stable [12]. Several predictors showed inconsistent results across studies, especially for demographic factors such as age and gender. These inconsistencies likely reflect multiple underlying factors including the following: population heterogeneity—studies included diverse patient populations with varying disease phenotypes, treatment histories, and geographic backgrounds; confounding variables—retrospective studies may have inadequately controlled for unmeasured confounders such as disease severity at withdrawal, concurrent medications, or selection bias; statistical power limitations—many individual studies were underpowered to detect demographic associations reliably; and methodological variations—differences in outcome definitions, follow-up protocols, and analytical methods may have affected predictor identification. The majority of observational studies further limits the ability to formulate causal relationships between predictors and outcomes. This high—and phenotype-specific—failure rate means that stopping thiopurines should never be regarded as routine housekeeping. Instead, clinicians must apply a “treat-to-target” philosophy in which withdrawal is considered only after deep remission has been documented endoscopically and biochemically, fully aligned with the STRIDE-II therapeutic-target framework [47].

In our review, relapse rates were comparable between monotherapy and combination therapy, although the latter analysis was limited by a smaller sample size. Roblin et al., (2017) reported that withdrawing azathioprine from combination therapy increased relapse risk and adversely affected infliximab pharmacokinetics, whereas dose reduction maintained therapeutic stability [48]. Similarly, Dohos et al., (2021) noted that discontinuation of immunomodulators in combination with biologics did not significantly elevate relapse risk compared with continuation [49]. Dohos et al., (2021) found that continued immunomodulator monotherapy is preferable in Crohn’s disease, while its withdrawal in ulcerative colitis did not significantly increase relapse risk within 24 months [49]. Consistent with these findings, a systematic review by Torres et al., (2015) reported high relapse rates after stopping immunomodulator monotherapy in both Crohn’s disease and ulcerative colitis, with approximately 75% relapsing within five years [46]. In Crohn’s disease, discontinuing the immunomodulator in combination therapy did not increase relapse risk, although 55–60% of patients relapsed within 24 months [46]. Interestingly, Boyapati et al., (2018) reported a significantly higher relapse rate in patients with quiescent Crohn’s disease who discontinued azathioprine monotherapy compared with those who continued treatment [50].

An open-label randomized trial showed that, in combination therapy, reducing the azathioprine dose—but not stopping it completely—was as effective as keeping the full dose [48]. A cohort study also found no meaningful differences in anti-TNF levels or in clinical and biological remission rates between different azathioprine doses [51]. In a population pharmacokinetic study, Lin et al., (2021) reported that body weight, TPMT3C genotype, and use of mesalazine each lowered 6-TGN clearance in adults with inflammatory bowel disease [52]. Their model suggests that TPMT3C carriers or patients taking mesalazine may need dose reduction to avoid high drug levels and toxicity [52].

Subgroup analysis revealed a significantly higher relapse rate in patients with ulcerative colitis (UC) compared with those with Crohn’s disease (CD) (41.3% vs. 24.7%, *p* < 0.05). The nearly two-fold higher relapse rate in UC observed in our meta-analysis is consistent with real-world experience and supports current guideline recommendations to continue the long-term use of thiopurines in UC when well tolerated [53,54]. A double-blind randomized controlled trial assessing azathioprine withdrawal in UC reported one-year relapse rates of 59% after withdrawal versus 36% with continued therapy, with the highest risk observed in patients with short-term remission (<6 months) [29].

Biomarkers of low-grade inflammation were consistently linked to subsequent flares, notably elevated C-reactive protein (CRP > 5–20 mg/L) and fecal calprotectin (>50–300 µg/g). Additional risk factors included shorter azathioprine (AZA) treatment duration (<4 years), absence of mucosal healing, and prior biologic therapy. While demographic variables such as age, sex, and smoking status generally showed limited and inconsistent associations, several studies reported higher relapse risk in male patients and those diagnosed at a younger age. However, female sex, younger age, and a prolonged time to remission (>6 months) were also associated with increased relapse risk in a previous cohort. A prior systematic review by Torres et al., (2015) identified multiple predictors of relapse after AZA withdrawal [46]. In Crohn’s disease, these included markers of active inflammation, poor prognostic features, and shorter remission duration [46]. In ulcerative colitis, relapse risk was linked to extensive disease, younger age, male sex, frequent prior relapses, and shorter AZA exposure. Overall, higher baseline disease activity and a complex disease course were consistently associated with future relapse [46]. Additionally, for Crohn’s patients, a model-based risk–benefit analysis of thiopurine withdrawal in CD patients in prolonged remission found that, in those without extensive colitis, continuation marginally increased life expectancy for 35-year-old men and women but decreased it for 65-year-old men and women. Based on this model, withdrawal became the preferred strategy at approximately 40 years of age for men and 45 years for women without extensive colitis, whereas in patients with extensive colitis, continuation was favored regardless of age [55]. Furthermore, pharmacogenetic insights from prior reviews suggest that NUDT153 and NUDT152 variants may help identify Asian patients at heightened risk of developing early thiopurine-induced myelotoxicity, underscoring the importance of individualized treatment strategies [56]. While biomarker predictors, including elevated CRP and fecal calprotectin, demonstrated directional associations across the included studies, the majority of the observational evidence base and significant between-study heterogeneity limit confidence in their predictive accuracy. These biomarkers should be considered as part of a detailed and structured risk assessment rather than definitive withdrawal criteria.

Meta-regression, as demonstrated in Figure 6, showed a clear dose–duration association: for every additional month of continuous azathioprine therapy, the risk of relapse fell by about 0.18 percentage points, explaining roughly one-third of the heterogeneity between studies. For patients with mild adverse effects or concerns about cumulative malignancy risk, dose reduction—shown in a randomized trial to preserve remission and biologic pharmacokinetics—may offer a pragmatic compromise [48]. Notably, Bouhnik et al., (1996) reported that, after four years of remission, relapse risk was similar regardless of whether therapy was continued or stopped, raising questions about the benefit of prolonged use in such cases [57]. Despite identifying AZA duration as a significant predictor, the meta-regression model explained only 31.2% of between-study variance, indicating substantial unmeasured heterogeneity. Possible sources include unmeasured disease severity markers, variations in withdrawal protocols, different outcome ascertainment methods, population-specific genetic factors, and healthcare system differences. This high residual variance severely limits the applicability of duration-based recommendations and highlights the need for individualized risk assessment.

Finally, decisions should be individualized through the shared discussion of competing risks. Continued azathioprine use lowers relapse and healthcare utilization—an important consideration in resource-constrained settings where the cost of a single flare may exceed that of several years of low-dose therapy—but must be weighed against small, cumulative risks of myelosuppression, non-melanoma skin cancer, and lymphoproliferative disease. Population pharmacokinetic data show that genotype-guided dose optimization (e.g., 50% reduction in TPMT*3C carriers) can mitigate toxicity without sacrificing efficacy, arguing for dose tailoring rather than reflex discontinuation in many patients [53]. In sum, azathioprine withdrawal should be reserved for carefully selected patients who have achieved deep, biomarker-negative remission, with extra caution in UC. Where doubts persist, dose reduction and close, biomarker-led monitoring provide a safer route than abrupt cessation, balancing the lifelong nature of IBD against the long-term toxicities of thiopurine therapy.

*Strengths and Limitations*

Our review has several notable strengths that reinforce its methodological rigor and relevance. First, it adhered closely to PRISMA 2020 guidelines and was prospectively registered in PROSPERO, ensuring transparency and reproducibility. Second, the search strategy was comprehensive and inclusive, capturing more than 3000 patients across diverse study designs, geographic regions, and clinical settings. Third, the robustness of the evidence synthesis was enhanced by performing a detailed risk of bias assessment, conducting subgroup analyses and sensitivity testing, and applying the GRADE framework to evaluate certainty of evidence. Collectively, these features strengthen the credibility of our findings and reflect a commitment to high-quality evidence synthesis.

At the same time, we recognize several important limitations that temper the interpretation of our results. The most prominent challenge was the substantial heterogeneity (I^2^ = 94.2%) observed across included studies. This heterogeneity likely reflects not only statistical variability but also important clinical and methodological differences, such as inconsistent definitions of clinical remission (ranging from standardized indices like CDAI < 150 or Mayo score ≤ 2, to less structured physician global assessments), variability in withdrawal protocols (gradual tapering versus abrupt discontinuation), and geographic variations in patient populations and clinical practices. Additionally, the predominance of observational studies and the limited degree of blinding within the available randomized trials inevitably introduce bias and reduce the strength of the pooled conclusions. The inconsistent reporting of key variables, including mucosal healing and biomarker outcomes, further restricts comparability and synthesis across studies. Taken together, these limitations underscore that, while the pooled estimates offer useful insights, they should be interpreted with caution when translated into clinical practice. Nevertheless, we believe the disease-specific comparison of relapse rates between ulcerative colitis and Crohn’s disease represents the most consistent and clinically meaningful finding of this review. Despite methodological challenges, this distinction provides an important contribution to the literature and may guide future clinical and research priorities. Finally, as with any meta-analysis, the potential for publication bias cannot be fully excluded.

*Future Directions*

Looking ahead, there is a clear need for prospective trials that explore AZA withdrawal in a more targeted way, especially by using biomarkers to better define who can stop treatment safely. These studies should ideally agree on what constitutes a relapse, use centralized endoscopic scoring, and include tools like genomics, microbiome data, and proteomics to create more personalized risk calculators. It would also be valuable to compare the long-term costs and benefits of continuing AZA, withdrawing it with monitoring, or starting biologics early—this kind of analysis can help shape future policy and insurance decisions. Lastly, genetic markers such as NUDT15 and TPMT variants deserve more attention, as they might influence how successful withdrawal can be for different patients.

## 5. Conclusions

Our study findings demonstrated low to very low certainty evidence that relapse rates after AZA withdrawal are significant but highly variable. The high heterogeneity and methodological limitations preclude universal application of these findings and underscore the need for individualized decision-making based on patient-specific factors. The GRADE assessment classified evidence quality as low to very low for most outcomes, reflecting serious concerns about study limitations, inconsistency, and imprecision. This level of evidence is insufficient to currently formulate definitive practice guidelines for AZA withdrawal decisions. Current evidence should inform but not dictate decision-making, which must include individual patient factors, clinical judgment, and shared decision-making principles.

## Figures and Tables

**Table 1:** Included studies’ characteristics, demographics, and treatment protocols.

Study	Design	Country	Setting	Follow-Up	Number	CD/UC	Mean Age	Male (%)	Disease Duration	AZA Duration	Withdrawal Method	Withdrawal Reason	Mono/Combo (Number)
Angelucci et al., 2010 [24]	Retrospective cohort	Italy	NR	≥1 yr	41	41/0	NR	NR	NR	NR	Abrupt	NR	41/0
Cassinotti et al., 2009 [25]	Retrospective cohort	Italy	Multicenter	Median 55 mo	127	0/127	38 yrs	60%	Median 5 yrs	Median 47 mo	Abrupt	Elective choice/toxicity	127/0
Cassinotti et al., 2021 [26]	Prospective cohort	Italy	Single center	Median 50 mo	57	26/31	49 yrs (UC), 45 yrs (CD)	65% (UC), 50% (CD)	Mean 16 yrs	Median 7 yrs	Abrupt	Extended deep remission	57/0
Crepaldi et al., 2023 [27]	Retrospective cohort	Italy	Single center	Median 3.5 yrs	274	141/133	29–33 yrs	57%	NR	NR	Abrupt	Remission/inefficacy/side effects	69/0
Fraser et al., 2002 [28]	Retrospective cohort	UK	Single center	Mean 6.9 yrs	222	79/143	NR	NR	NR	Mean 1.7 yrs	Abrupt	Remission	222/0
Hawthorne et al., 1992 [29]	RCT	UK	5 hospitals	12 mo	67	2/65	44 yrs	50.7%	Mean 8 yrs	Median 20 mo	Abrupt (randomized)	Randomized withdrawal	67/0
Holtmann et al., 2006 [30]	Retrospective cohort	Europe	16 centers	NR	1176	818/358	23–28 yrs	48.5%	Median 3.8–5.9 yrs	NR	Abrupt	Physician/patient decision	1176/0
Iborra et al., 2019 [31]	Multicenter observational	Spain	3 hospitals	Median 36.7 mo	95	60/35	48.6 yrs (CD), 51.9 yrs (UC)	43.3% (CD), 60% (UC)	NR	Median 77–87 mo	Abrupt	Physician discretion	95/0
Jorissen et al., 2021 [32]	Retrospective cohort	Belgium	Single center	Median 66 mo	91	55/36	>60 yrs	60%	NR	Median 82.5 mo	Abrupt	Physician decision due to age	76/15
Kennedy et al., 2014 [33]	Retrospective cohort	UK	Multicenter	Median 34 mo	237	129/108	38 yrs (CD), 42 yrs (UC)	48.5%	NR	Median 6.0 yrs	Tapered in 37%	Physician decision	237/0
Lémann et al., 2005 [16]	RCT	France and Belgium	12 centers	18 mo	83	83/0	~38 yrs	44.6%	Median 11 yrs	Median 65.5 mo	Abrupt (randomized)	Randomized withdrawal	83/0
Louis et al., 2023 [34]	RCT	Europe and Australia	64 hospitals	104 weeks	207	207/0	31–36 yrs	57%	Median 6.4–6.8 yrs	Median 2.3–2.6 yrs	Abrupt (randomized)	Randomized withdrawal	71/136
Moreno-Rincón et al., 2015 [35]	Retrospective cohort	Spain	Multicenter	Median 27 mo	102	0/102	32 yrs	46.1%	Median 12 yrs	Median 51 mo	Abrupt	Physician/patient decision	102/0
Nyman et al., 1985 [36]	Retrospective cohort	Sweden	Single center	Mean 5.75 yrs	42	42/0	26 yrs	47.6%	Mean 5.1 yrs	Mean 4.1 yrs	Abrupt	Pancreatitis	38/4
O’Donoghue et al., 1978 [37]	RCT	UK	Multicenter	12 mo	51	51/0	~40 yrs	43.1%	Mean 7.6 yrs	NR	Abrupt (randomized)	Randomized withdrawal	36/15
Oussalah et al., 2010 [38]	Retrospective cohort	France	Single center	Mean 14 mo	48	48/0	27 yrs	52%	Median 6.7 yrs	Median 30.2 mo	Abrupt	Physician decision	0/48
Ranjan et al., 2022 [39]	Retrospective cohort	India	Single center	Median 25 mo	218	39/179	30.2 yrs	64.7%	Median 9.6 yrs	Median 30 mo	Abrupt	NR	218/0
Sokol et al., 2010 [40]	Retrospective cohort	France	Single center	>24 mo	47	47/0	NR	57%	NR	Median 58.7 mo	Abrupt	Personal convenience	47/0
Treton et al., 2009 [41]	Prospective cohort	France and Belgium	Multicenter	Median 54.5 mo	66	66/0	37 yrs	44%	Median 10.2 yrs	Median 68.4 mo	Abrupt	Medical/personal decision	66/0
Van Assche et al., 2008 [42]	RCT	Belgium	Multicenter	104 weeks	80	80/0	~35 yrs	45%	Median 9 yrs	Median 30 mo	Abrupt (randomized)	Randomized withdrawal	0/80
Vilien et al., 2004 [43]	RCT	Denmark	Multicenter	12 mo	29	29/0	~40 yrs	NR	NR	Median 37 mo	Abrupt (randomized)	Randomized withdrawal	29/0
Wenzl et al., 2015 [44]	RCT	Austria	Two-center	24 mo	52	52/0	39 yrs	44.2%	Median 9 yrs	Median 5.2 yrs	Abrupt (randomized)	Randomized withdrawal	52/0

Abbreviations: AZA, azathioprine; CD, Crohn’s disease; UC, ulcerative colitis; RCT, randomized controlled trial; NR, not reported; mo, months; yrs, years; Mono, monotherapy; Combo, combination therapy.

**Table 2:** Disease activity at withdrawal and relapse outcomes.

Study	Number	Disease Activity at Withdrawal	Steroid-Free (Number)	Mucosal Healing (Number)	Total Relapses (Number)	Overall Relapse Rate (%)	UC Relapse Rate	CD Relapse Rate	12-Month Relapse Rate	24-Month Relapse Rate	Median Time to Relapse
Angelucci et al., 2010 [24]	41	CDAI < 150	NR	NR	27	65.9%	N/A	65.9%	NR	NR	NR
Cassinotti et al., 2009 [25]	127	Steroid-free remission	127	NR	85	67%	67%	N/A	35%	49%	12 mo
Cassinotti et al., 2021 [26]	57	CDAI < 150 (CD), Mayo < 2 (UC), CRP ≤ 10 mg/L, FC ≤ 50 µg/g	57	57 (100%)	26	46%	58%	31%	NR	NR	15 mo
Crepaldi et al., 2023 [27]	274	NR	274	NR	21	30.4% (remission group)	NR	NR	11%	21%	NR
Fraser et al., 2002 [28]	222	NR	222	NR	NR	~63%	NR	NR	37%	NR	NR
Hawthorne et al., 1992 [29]	67	Grade 0–1 sigmoidoscopy	67	67 (100%)	32	47.8%	47.8%	NR	59% (placebo), 36% (AZA)	NR	NR
Holtmann et al., 2006 [30]	1176	NR	NR	NR	NR	NR	NR	NR	NR	NR	NR
Iborra et al., 2019 [31]	95	CDAI < 150 (CD), Mayo ≤ 2 (UC), normal CRP/FC	95	58/95 (61%)	26	27.4%	26%	28%	UC: 23.4%, CD: 12.9%	NR	UC: 36.3 mo, CD: 38.5 mo
Jorissen et al., 2021 [32]	91	CRP < 5.0 mg/L	91	37/91 (40.7%)	28	30.8%	36%	27%	~10%	~20%	21 mo
Kennedy et al., 2014 [33]	237	CDAI < 150 (CD), By PGA (UC)	237	NR	NR	UC: 12%, CD: 23% at 12 mo	12%	23%	UC: 12%, CD: 23%	UC: 26%, CD: 39%	NR
Lémann et al. 2005 [16]	83	CDAI < 150	78/83	19/45 with ulcers	12	14.5%	N/A	14.5%	NR	21% (placebo), 8% (AZA) at 18 mo	NR
Louis et al., 2023 [34]	207	CDAI < 150	207	22/207 with ulcers	39	18.8%	N/A	18.8%	NR	14% (combo), 36% (IFX withdrawal)	NR
Moreno-Rincón et al., 2015 [35]	102	Normal stools, no blood/pus, normal CRP	102	22/102 (21.6%)	33	32.4%	32.4%	N/A	18.9%	NR	12 mo
Nyman et al., 1985 [36]	42	NR	42	NR	1	2.6% (AZA group)	N/A	2.6%	NR	NR	NR
O’Donoghue et al., 1978 [37]	51	Remission or stable good health	51	NR	10	19.6%	N/A	19.6%	41% (placebo), 5% (AZA)	NR	NR
Oussalah et al., 2010 [38]	48	CDAI < 150, normal CRP	48	NR	13	27%	N/A	27%	15%	59%	NR
Ranjan et al., 2022 [39]	218	CDAI < 150 (CD), Mayo ≤ 2 (UC)	218	NR	80	36.7%	39.7%	23%	17%	NR	20 mo
Sokol et al., 2010 [40]	47	CDAI < 150	NR	NR	NR	NR	N/A	NR	NR	57.3%	NR
Treton et al., 2009 [41]	66	CDAI < 150, HBI < 4, Normal CRP (62/66)	61/66	NR	32	48.5%	N/A	48.5%	14%	NR	NR
Van Assche et al., 2008 [42]	80	CDAI < 150	80	30/49 (61%)	22	55%	N/A	55%	NR	NR	NR
Vilien et al., 2004 [43]	29	Inactive disease	29	NR	10	34.5%	N/A	34.5%	53% (withdrawal), 15% (AZA)	NR	NR
Wenzl et al., 2015 [44]	52	CDAI < 150	52	10/28 with ulcers	12	23.1%	N/A	23.1%	24% (placebo), 4% (AZA)	32% (placebo), 14% (AZA)	NR

Abbreviations: CDAI, Crohn’s Disease Activity Index; Mayo, Mayo score; HBI, Harvey–Bradshaw Index; CRP, C-reactive protein; FC, fecal calprotectin; PGA, physician global assessment; IFX, infliximab; AZA, azathioprine; UC, ulcerative colitis; CD, Crohn’s disease; mo, months; NR, not reported; N/A, not applicable.

**Table 3:** Time-to-event and disease predictors.

Study	Median Time to Relapse	Cumulative Relapse Rates	Age Predictor	Gender Predictor	Disease Type Predictor	Smoking Predictor	Prior Surgery Predictor	Other Predictors
Angelucci et al., 2010 [24]	NR	NR	No	No	NR	Yes (current smoking protective)	NR	NR
Cassinotti et al., 2009 [25]	12 mo (range 1–119)	35% at 1 yr, 49% at 2 yrs, 65% at 5 yrs	No	Yes (male)	No	NR	NR	Extensive colitis, shorter AZA duration, withdrawal due to toxicity
Cassinotti et al., 2021 [26]	15 mo (UC and CD)	NR	Yes (≤45 yrs for UC)	Yes (female for UC)	No	NR	NR	Fecal calprotectin for both CD and UC
Crepaldi et al., 2023 [27]	NR	11% at 1 yr, 21% at 2 yrs	Yes (older >35 yrs had lower relapse risk)	No	No	NR	NR	Reason for stopping (remission vs. active)
Fraser et al., 2002 [28]	NR	37% at 1 yr, 66% at 3 yrs, 75% at 5 yrs	Yes (Older age protective)	No	No	NR	NR	Lower WBC or neutrophil count, higher MCV
Hawthorne et al., 1992 [29]	NR	Placebo: 59% at 1 yr; AZA: 36% at 1 yr	Yes (older age protective)	No	NR	NR	NR	Mucosal healing
Holtmann et al., 2006 [30]	NR	NR	No	No	No	NR	NR	NR
Iborra et al., 2019 [31]	36.3 mo (UC), 38.5 mo (CD)	CD: 12.9% at 1 yr, 46.7% at 5 yrs; UC: 23.4% at 1 yr, 46.2% at 5 yrs	No	No	No	NR	NR	Corticosteroid dependence, early AZA start (CD), late AZA start (UC)
Jorissen et al., 2021 [32]	21 mo	NR	No	No	No	NR	NR	Shorter duration of AZA therapy
Kennedy et al., 2014 [33]	NR	CD: 23% at 12 mo, 39% at 24 mo; UC: 12% at 12 mo, 26% at 24 mo	No	No	Yes (CD > UC)	NR	NR	Elevated CRP in CD, elevated WCC in UC, tapering at withdrawal (CD)
Lémann et al. 2005 [16]	NR	Placebo: 21% at 18 mo; AZA: 8% at 18 mo	No	No	NR	NR	NR	CRP > 20 mg/L, Hb < 12 g/dL, time without steroids < 50 mo
Louis et al., 2023 [34]	NR	Combo: 14% at 2 yrs, AZA withdrawal: 10% at 2 yrs, IFX withdrawal: 36% at 2 yrs	Yes (<17 yrs at diagnosis)	No	NR	NR	NR	Higher CDEIS, 6-TGN > 300 protective in IFX withdrawal, hsCRP, FC > 300
Moreno-Rincón et al., 2015 [35]	12 mo (IQR: 7–24)	18.9% at 12 mo, 36.5% at 36 mo, 43% at 5 yrs	No	No	No	NR	NR	Longer remission duration protective, biological remission protective, pancolitis
Nyman et al., 1985 [36]	NR	26% in complete remission	No	No	NR	NR	NR	NR
O’Donoghue et al., 1978 [37]	NR	Placebo: 25% at 6 mo, 41% at 1 yr; AZA: 0% at 6 mo, 5% at 1 yr	No	No	NR	NR	NR	NR
Oussalah et al., 2010 [38]	NR	15% at 12 mo, 59% at 24 mo	No	No	NR	NR	NR	CRP > 5 mg/L, platelets > 298, shorter combo therapy duration (≤27 mo)
Ranjan et al., 2022 [39]	20 mo (IQR: 9–49)	17% at 1 yr, 34% at 3 yrs, 44% at 5 yrs	No	Yes (male, HR 1.6)	No	NR	NR	Shorter duration of AZA therapy (HR 1.02)
Sokol et al., 2010 [40]	NR	57.3% at 2 yrs, 73.3% at 5 yrs	No	Yes (male, OR 2.42)	NR	Yes (absence of smoking, OR 2.78)	NR	NR
Treton et al., 2009 [41]	NR	14% at 1 yr, 52.8% at 3 yrs, 62.7% at 5 yrs	No	No	NR	NR	NR	CRP ≥ 20 mg/L, Hb < 12 g/dL, neutrophils ≥ 4 × 10^9^/L
Van Assche et al., 2008 [42]	NR	NR	No	No	NR	No (smoking, disease location, type of IS not predictive)	NR	NR
Vilien et al., 2004 [43]	NR	Withdrawal: 53% at 1 yr; AZA: 15% at 1 yr	No	No	NR	NR	NR	NR
Wenzl et al., 2015 [44]	NR	Placebo: 24% at 1 yr, 32% at 2 yrs; AZA: 4% at 1 yr, 14% at 2 yrs	No	No	NR	NR	NR	Higher AZA dose at enrollment

Abbreviations: HR, hazard ratio; OR, odds ratio; UC, ulcerative colitis; CD, Crohn’s disease; AZA, azathioprine; CRP, C-reactive protein; FC, fecal calprotectin; WCC, white cell count; Hb, hemoglobin; MCV, mean corpuscular volume; CDEIS, Crohn’s Disease Endoscopic Index of Severity; 6-TGN, 6-thioguanine nucleotide; hsCRP, high-sensitivity C-reactive protein; IFX, infliximab; mo, months; yr, year; NR, not reported.

**Table 4:** Assessment and evaluation of relapse predictors after azathioprine withdrawal from included Studies.

Study	Number	Disease	Biomarker Predictors (Cut-Off, Effect)	Clinical/Demographic Predictors (Effect)	Treatment/Disease-Specific Predictors (Effect)	Protective Factors	Risk Factors	Multivariate Analysis	*p*-Value
Cassinotti et al., 2009 [25]	127	UC	None evaluated systematically	Male gender (risk), extensive colitis vs. limited (risk)	Shorter AZA duration (risk), withdrawal due to toxicity (risk), 47 mo median duration	Female gender, limited colitis, longer AZA therapy	Male gender, extensive colitis, short AZA duration, Toxicity withdrawal	Yes	Not specified
Cassinotti et al., 2021 [26]	57	CD + UC	CRP ≤ 10 mg/L baseline → elevated CRP (UC: HR 4.1, *p* = 0.02; CD: NS), FC ≤ 50 μg/g baseline → elevated FC (UC: HR 3.3, *p* = 0.03; CD: HR 4.5, *p* = 0.025)	Age ≤ 45 yrs (UC risk), female gender (UC risk), male gender (CD: NS)	100% mucosal healing at baseline, deep remission criteria	Mucosal healing, older age > 45 (UC), male gender (UC)	Elevated CRP (UC), elevated FC (both), Young age ≤ 45 (UC), female (UC)	Yes	*p* = 0.02–0.03
Crepaldi et al., 2023 [27]	274	CD + UC	None systematically evaluated	Older age > 35 yrs (protective)	Withdrawal reason: remission vs. active disease/side effects	Age > 35 yrs, withdrawal in remission	Age ≤ 35 yrs, withdrawal for active disease/AEs	Yes	Not specified
Fraser et al., 2002 [28]	222	CD + UC	Lower WBC (protective), lower neutrophils (protective), higher MCV (protective)	Older age (protective), gender (NS)	1.7 years mean AZA duration, remission-based withdrawal	Older age, lower WBC/neutrophils, higher MCV	Younger age, higher WBC/neutrophils, lower MCV	Partial	Not specified
Hawthorne et al., 1992 [29]	67	UC	None systematically evaluated	Older age (protective), gender (NS)	Mucosal healing grade 0–1 (protective), 20 mo median AZA, RCT design	Older age, mucosal healing grade 0–1	Younger age, active endoscopy	Yes	Not specified
Iborra et al., 2019 [31]	95	CD + UC	Normal CRP/FC at baseline → FC levels at 1 year (UC: *p* = 0.03, CD: NS)	Corticosteroid dependence (HR 3.18), age (NS), gender (NS)	Early AZA start protective (CD), late AZA start risk (UC), AZA duration 77–87 mo	Early AZA initiation (CD), steroid independence	Corticosteroid dependence, late AZA start (UC), FC elevation at 1 yr (UC)	Yes	*p* = 0.03
Jorissen et al., 2021 [32]	91	CD + UC	CRP < 5.0 mg/L baseline (inclusion), no predictive biomarkers identified	Elderly population > 60 yrs, gender (NS)	Shorter AZA duration 82.5 mo median (risk), age-related withdrawal	Longer AZA duration	Shorter AZA treatment duration	Partial	Not specified
Kennedy et al., 2014 [33]	237	CD + UC	Elevated CRP (CD predictor), elevated WCC (UC predictor), normal values protective	Disease type (CD > UC, *p* = 0.035), age (NS), gender (NS)	Tapering withdrawal (CD), physician decision trigger, 6-year median AZA duration	Normal CRP/WCC, UC disease type, abrupt withdrawal	CD disease type, elevated CRP (CD), elevated WCC (UC), tapering (CD)	Yes	*p* = 0.035
Lémann et al. 2005 [16]	83	CD	CRP > 20 mg/L (risk), Hb < 12 g/dL (risk), normal values protective	Age ~38 yrs (NS), gender (NS)	Time without steroids < 50 mo (risk), 65.5 mo median AZA, RCT withdrawal	CRP ≤ 20, Hb ≥ 12, steroid-free > 50 mo	CRP > 20, anemia, short steroid-free period	Yes	Not specified
Louis et al., 2023 [34]	207	CD	hsCRP (elevated), FC > 300 μg/g (risk), 6-TGN > 300 pmol/8 × 10^8^ RBC (protective in IFX withdrawal)	Age < 17 yrs at diagnosis (risk), male gender (NS)	Higher CDEIS score (risk), IFX vs. AZA withdrawal strategy	6-TGN > 300, continued combination therapy	Young age < 17 yrs, higher CDEIS, FC > 300	Yes	Various
Moreno-Rincón et al., 2015 [35]	102	UC	Biological remission status (protective): CRP normal + FC normal + endoscopic healing	Pancolitis E3 extent (risk), age 32 yrs (NS), gender (NS)	Longer remission duration > 33 mo (protective), 51 mo median AZA, physician/patient decision	Biological remission, longer remission > 33 mo, limited extent	Pancolitis extent, shorter remission duration	Yes	Not specified
Oussalah et al., 2010 [38]	48	CD	CRP > 5 mg/L (risk), platelets > 298 × 10^9^/L (risk), normal ranges protective	Age 27 yrs (NS), male 52% (NS)	Shorter combo therapy ≤ 27 mo (risk), IFX + AZA combination, physician decision	Longer combination therapy > 27 mo, normal CRP ≤ 5	Short combination duration ≤ 27 mo, CRP > 5, platelets > 298	Yes	Not specified
Ranjan et al., 2022 [39]	218	CD + UC	CRP (NS), FC (NS)	Male gender (HR 1.6, 95% CI NR), age (NS), disease type (NS)	AZA duration (HR 1.02 per month shorter, 95% CI NR), monotherapy only	Longer AZA duration, female gender	Male gender, shorter AZA duration < 4 yrs	Yes	*p* < 0.05
Sokol et al., 2010 [40]	47	CD	None significant	Male gender (OR 2.42, 95% CI NR), Non-smoking status (OR 2.78, 95% CI NR)	AZA duration 58.7 mo (NS), personal convenience withdrawal	Current smoking, female gender	Male gender, non-smoking status	Yes	Not specified
Treton et al., 2009 [41]	66	CD	CRP ≥ 20 mg/L (risk), Hb < 12 g/dL (risk), neutrophils ≥ 4 × 10^9^/L (risk), normal values protective	Age 37 yrs (NS), male 44% (NS)	AZA duration 68.4 mo (NS), steroid-free status, medical/personal decision	Normal CRP < 20, Hb ≥ 12, neutrophils < 4 × 10^9^	CRP ≥ 20, Anemia Hb < 12, neutrophilia ≥ 4 × 10^9^	Yes	Not specified

Abbreviations: CRP, C-reactive protein; FC, fecal calprotectin; hsCRP, high-sensitivity C-reactive protein; HR, hazard ratio; OR, odds ratio; CI, confidence interval; UC, ulcerative colitis; CD, Crohn’s disease; AZA, azathioprine; IFX, infliximab; 6-TGN, 6-thioguanine nucleotide; CDEIS, Crohn’s Disease Endoscopic Index of Severity; WCC, white cell count; Hb, hemoglobin; MCV, mean corpuscular volume; RBC, red blood cells; NS, not significant; RCT, randomized controlled trial; AEs, adverse events; yrs, years; mo, months.

**Table 5:** Disease subgrouping and post-relapse management outcomes.

Study	Number	Disease Phenotype Subgroups	Age/Gender Subgroups	Relapse Severity	Hospitalization (Number)	Surgery (Number)	Rescue Therapy	AZA Reintroduction	Biologic Escalation	Response to Retreatment	Long-term Outcomes
Angelucci et al., 2010 [24]	41	CD only: Limited data	NR	NR	NR	NR	NR	NR	NR	NR	Abstract with limited data
Cassinotti et al., 2009 [25]	127	UC only: Extensive colitis increased risk vs. limited	Male gender increased risk; age 38 yrs at AZA start	NR	NR	NR	Rescue therapy (CS, CsA, colectomy)	NR	NR	NR	67% relapse rate—high risk population
Cassinotti et al., 2021 [26]	57	CD (26): Ileal 46%, ileocolonic 39%, colonic 15%; B1 38%, B2 54%, B3 8%; UC (31): left-sided 29%, extensive 71%	UC: Female risk factor, age ≤ 45 yrs risk; CD: Gender NS	UC: 56% moderate severity; CD: All mild severity	0	0	UC: 2 steroids, 9 AZA, 5 anti-TNF; CD: 5 AZA, 4 anti-TNF	UC: 9/18; CD: 5/8	UC: 5/18; CD: 4/8	AZA: UC 7/9, CD 4/5; anti-TNF: UC 5/5, CD response NR	No hospitalizations or surgery
Crepaldi et al., 2023 [27]	274	CD (141): L1 30%, L2 20%, L3 50%; B1 43.3%, B2 35%, B3 21%; UC (133): E1 12%, E2 28.6%, E3 57.1%	Age > 35 yrs protective; gender: Male 57% vs. female 43%	NR	NR	NR	NR	NR	NR	NR	Focus on withdrawal reasons
Fraser et al., 2002 [28]	222	CD (79) vs. UC (143): Large cohort, limited phenotype analysis	Older age protective; gender NR	NR	NR	NR	NR	NR	NR	NR	Long-term follow-up, limited detail
Hawthorne et al., 1992 [29]	67	UC only: Sigmoidoscopy grade 0–1 required	Older age protective, male 50.7%	Symptom and sigmoidoscopic deterioration	NR	NR	Change in treatment	NR	NR	NR	RCT design with endoscopic endpoint
Holtmann et al., 2006 [30]	1176	CD (818) vs. UC (358): Large cohort but limited subgroup analysis	Age 23–28 yrs at AZA start, male 48.5%	NR	NR	NR	Oral steroid dosage increases	NR	NR	NR	Large cohort, limited outcome detail
Iborra et al., 2019 [31]	95	CD (60): L1 30%, L2 20%, L3 50%; UC (35): E1 12%, E2 28.6%, E3 57.1%	No age/gender subgroup differences in relapse	NR	NR	NR	Various rescue therapies	UC: 9, CD: 10	NR	UC: 4/9 (44%); CD: 10/10 (100%)	Superior re-treatment response in CD
Jorissen et al., 2021 [32]	91	CD (55) vs. UC (36): No specific Montreal subgroup analysis	Elderly cohort > 60 yrs; male 60%	NR	NR	10 total (2 UC colectomy, 8 CD resections)	17 patients (steroids + biologics)	1	17	NR	Cancer: 26 patients, Mortality: 6 patients
Kennedy et al., 2014 [33]	237	CD (129) vs. UC (108): Disease type main predictor (CD > UC relapse risk)	CD vs. UC: Age 38 vs. 42 yrs; overall Male 48.5%	Moderate-to-severe relapse definition	Hospital admission as outcome	Surgery as outcome	Oral steroids, thiopurine recommencement	Thiopurine recommencement	Anti-TNF as outcome	NR	Comprehensive outcome tracking
Lémann et al., 2005 [16]	83	CD only: Phenotype not analyzed as subgroups	Age ~38 yrs, male 44.6%; no age/gender effects	Defined by CDAI criteria	NR	Surgery as endpoint	Re-treatment per protocol	NR	NR	NR	RCT with defined endpoints
Louis et al., 2023 [34]	207	CD only: Disease location/behavior not specified as subgroup analysis	Age < 17 yrs at diagnosis (risk factor); male 57%	NR	NR	NR	Step-up approach based on relapse severity	25 patients (35.2% of IFX withdrawal group)	NR	Response rates not specified	Primary endpoint: relapse-free survival
Moreno-Rincón et al., 2015 [35]	102	UC only: E1 vs. E2 vs. E3—Pancolitis (E3) increased risk	Age 32 yrs, Male 46.1%; no age/gender effects	NR	NR	NR	Rescue therapy as per relapse	NR	NR	NR	Focus on remission duration effects
Nyman et al., 1985 [36]	42	CD only: Small historical cohort	Age 26 yrs at AZA start, male 47.6%	Clinical deterioration	NR	NR	NR	NR	NR	NR	Historical study, limited data
O’Donoghue et al., 1978 [37]	51	CD only: Historical RCT	Age ~40 yrs, male 43.1%	Significant deterioration requiring treatment change	NR	NR	Change in treatment	NR	NR	NR	Historical RCT
Oussalah et al., 2010 [38]	48	CD only: L1 29%, L2 50%, L3 21%, L4 4%; B1 71%, B2 21%, B3 8%	Pediatric onset 33% (<16 yrs); Male 52%	NR	NR	1	Treatment escalation	0	1 (Adalimumab)	NR	Low surgery rate in combination therapy
Ranjan et al., 2022 [39]	218	CD (39): Ileal 33.3%, Colonic 10.3%, ileocolonic 30.8%; B1 61.5%, B2 30.8%, B3 7.7%; UC (179): E1 2.8%, E2 41.9%, E3 55.3%	Male gender HR 1.6; UC vs. CD relapse: 39.7% vs. 23%	NR	13	6	Medical therapy escalation	NR	NR	NR	Surgery required in 6 patients
Sokol et al., 2010 [40]	47	CD only: Phenotype not analyzed as subgroups	Male OR 2.42 for relapse; non-smoking OR 2.78	NR	NR	NR	NR	NR	NR	NR	Focus on lifestyle factors
Treton et al., 2009 [41]	66	CD only: Phenotype not analyzed as subgroups	Age 37 yrs, male 44%; no age/gender effects	NR	NR	NR	Re-treatment as needed	NR	NR	NR	Focus on biomarker predictors
Van Assche et al., 2008 [42]	80	CD only: Smoking, disease location, IS type not predictive	Age ~35 yrs, Male 45%; no predictive value	Disease flare definition	NR	NR	Shortening IFX interval or stopping IFX	NR	Management per protocol	NR	RCT design limits management flexibility
Vilien et al., 2004 [43]	29	CD only: Small cohort	Age ~40 yrs; gender NR	Defined by CDAI and activity	NR	NR	Disease activity requiring intervention	NR	NR	NR	Small RCT
Wenzl et al., 2015 [44]	52	CD only: Phenotype subgroups not analyzed	Age 39 yrs, male 44.2%; no age/gender effects	NR	NR	NR	Placebo group: various; AZA continued group: maintained	NR	NR	NR	RCT design limits real-world management

Abbreviations: CD, Crohn’s disease; UC, ulcerative colitis; L1/L2/L3/L4, Montreal location classification; B1/B2/B3, Montreal behavior classification; E1/E2/E3, Montreal extent classification; AZA, azathioprine; Anti-TNF, anti-tumor necrosis factor; CS, corticosteroids; CsA, cyclosporine; IFX, infliximab; IS, immunosuppressant; CDAI, Crohn’s Disease Activity Index; RCT, randomized controlled trial; HR, hazard ratio; OR, odds ratio; NR, not reported; NS, not significant; yrs, years.

## Data Availability

All data generated or analyzed during this study are included in this published article.

## Supplementary Materials

The following supporting information can be downloaded at https://www.mdpi.com/article/10.3390/jcm14196868/s1, Table S1: PRISMA-2020 checklist; Table S2: Search strategy used in each of the databases; Table S3: Risk of bias assessment; Table S4: Certainty assessment of evidence.

## Abbreviations

The following abbreviations are used in this manuscript:

CD	Crohn’s disease
UC	Ulcerative colitis
L1/L2/L3/L4	Montreal Location Classification
B1/B2/B3	Montreal Behavior Classification
E1/E2/E3	Montreal Extent Classification
AZA	Azathioprine
Anti-TNF	Anti-tumor necrosis factor
CS	Corticosteroids
CsA	Cyclosporine
IFX	Infliximab
IS	Immunosuppressant
CDAI	Crohn’s Disease Activity Index
CDEIS	Crohn’s Disease Endoscopic Index of Severity (Endoscopic score for Crohn’s disease severity)
Mayo	Mayo score
HBI	Harvey–Bradshaw Index
PGA	Physician global assessment
CRP	C-reactive protein
hsCRP	High-sensitivity C-reactive protein
FC	Fecal calprotectin
WCC	White cell count
Hb	Hemoglobin
MCV	Mean corpuscular volume
RBC	Red blood cells
6-TGN	6-thioguanine nucleotide (active metabolite of thiopurines used to monitor therapy)
RCT	Randomized control trial
HR	Hazard ratio
OR	Odds ratio
CI	Confidence interval
AEs	Adverse events
NR	Not reported
NS	Not significant
N/A	Not applicable
Yr	Years
mo	Months
Mono	Monotherapy
Combo	Combination therapy
