# Supplementary material for: Relapse Rates and Predictors Following Azathioprine Withdrawal in Inflammatory Bowel Disease: A Systematic Review, Meta-Analysis, and Meta-Regression
Source: J Clin Med. 2025 Sep 28;14(19):6868. doi: 10.3390/jcm14196868 (PMC12524840; doi:10.3390/jcm14196868)
Supplement: Supplementary file 1 [file jcm-14-06868-s001.zip › jcm-3863434-supplementary.pdf]

Table S1. Search strategy used in each of the databases.

**Database**	**Search Strategy Used**
PubMed	(inflammatory bowel disease[MeSH] OR IBD OR Crohn's disease[MeSH] OR ulcerative colitis OR colitis) AND (azathioprine[MeSH] OR Imuran OR Immuran OR Imurel OR azathioprine sulfate OR azathioprine sodium OR "sodium, azathioprine" OR azathioprine sodium salt) AND (withdrawal OR discontinuation OR cessation OR stopping OR interruption OR relapse OR flare-up OR recurrence)
Embase	Embase <1974 to 2025 Week 01><br><br>1 inflammatory bowel disease.mp. 132312<br>2 IBD.mp. 84334<br>3 Crohn's disease.mp. 99209<br>4 ulcerative colitis.mp. 114454<br>5 colitis.mp. 190700<br>6 1 or 2 or 3 or 4 or 5 301709<br>7 azathioprine.mp. 118151<br>8 Imuran.mp. 2748<br>9 Imurel.mp. 613<br>10 azathioprine sulfate.mp. 1<br>11 sodium, azathioprine.mp. 3<br>12 7 or 8 or 9 or 10 or 11 118212<br>13 withdrawal.mp. 440229<br>14 discontinuation.mp. 130662<br>15 cessation.mp. 160767<br>16 stopping.mp. 43873<br>17 interruption.mp. 51873<br>18 relapse.mp. 361103<br>19 flare-up.mp. 3807<br>20 recurrence.mp. 834248<br>21 13 or 14 or 15 or 16 or 17 or 18 or 19 or 20 1730748<br>22 6 and 12 and 21 5773
Scopus	(inflammatory bowel disease OR IBD OR Crohn's disease OR ulcerative colitis OR colitis) AND (azathioprine OR Imuran OR Immuran OR Imurel OR azathioprine sulfate OR azathioprine sodium OR "sodium, azathioprine" OR azathioprine sodium salt) AND (withdrawal OR discontinuation OR cessation OR stopping OR interruption Or relapse OR flare-up OR recurrence)
Web of Science	TS = ("inflammatory bowel disease" OR IBD OR "Crohn's disease" OR "ulcerative colitis" OR colitis) AND TS = ("azathioprine" OR Imuran OR Immuran OR Imurel OR "azathioprine sulfate" OR "azathioprine sodium" OR "sodium, azathioprine" OR "azathioprine sodium salt") AND TS = ("withdrawal" OR "discontinuation" OR "cessation" OR "stopping" OR "interruption" OR "relapse" OR "flare-up" OR "recurrence")

Table S2. PRISMA 2020 Checklist

Section and Topic	Item #	Checklist item	Location where item is reported
**TITLE**			
Title	1	Identify the report as a systematic review.	Lines 2-4
**ABSTRACT**			
Abstract	2	See the PRISMA 2020 for Abstracts checklist.	Lines 22-43
**INTRODUCTION**			
Rationale	3	Describe the rationale for the review in the context of existing knowledge.	Lines 83-90
Objectives	4	Provide an explicit statement of the objective(s) or question(s) the review addresses.	Lines 87-90
**METHODS**			
Eligibility criteria	5	Specify the inclusion and exclusion criteria for the review and how studies were grouped for the syntheses.	Lines 126-137
Information sources	6	Specify all databases, registers, websites, organisations, reference lists and other sources searched or consulted to identify studies. Specify the date when each source was last searched or consulted.	Lines 108-124
Search strategy	7	Present the full search strategies for all databases, registers and websites, including any filters and limits used.	Lines 111-119 + Supplementary Material – 2 (Table S1)
Selection process	8	Specify the methods used to decide whether a study met the inclusion criteria of the review, including how many reviewers screened each record and each report retrieved, whether they worked independently, and if applicable, details of automation tools used in the process.	Lines 139-147
Data collection process	9	Specify the methods used to collect data from reports, including how many reviewers collected data from each report, whether they worked independently, any processes for obtaining or confirming data from study investigators, and if applicable, details of automation tools used in the process.	Lines 149-158
Data items	10a	List and define all outcomes for which data were sought. Specify whether all results that were compatible with each outcome domain in each study were sought (e.g. for all measures, time points, analyses), and if not, the methods used to decide which results to collect.	Lines 149-158
	10b	List and define all other variables for which data were sought (e.g. participant and intervention characteristics, funding sources). Describe any assumptions made about any missing or unclear information.	Lines 149-158
Study risk of bias assessment	11	Specify the methods used to assess risk of bias in the included studies, including details of the tool(s) used, how many reviewers assessed each study and whether they worked independently, and if applicable, details of automation tools used in the process.	Lines 160-174
Effect measures	12	Specify for each outcome the effect measure(s) (e.g. risk ratio, mean difference) used in the synthesis or presentation of results.	Lines 149-158
Synthesis methods	13a	Describe the processes used to decide which studies were eligible for each synthesis (e.g. tabulating the study intervention characteristics and comparing against the planned groups for each synthesis (item #5)).	Lines 126-137
	13b	Describe any methods required to prepare the data for presentation or synthesis, such as handling of missing	Lines 209-220

Section and Topic	Item #	Checklist item	Location where item is reported
		summary statistics, or data conversions.	
	13c	Describe any methods used to tabulate or visually display results of individual studies and syntheses.	Lines 149-158, 176-195
	13d	Describe any methods used to synthesize results and provide a rationale for the choice(s). If meta-analysis was performed, describe the model(s), method(s) to identify the presence and extent of statistical heterogeneity, and software package(s) used.	Lines 176-195
	13e	Describe any methods used to explore possible causes of heterogeneity among study results (e.g. subgroup analysis, meta-regression).	Lines 197-220
	13f	Describe any sensitivity analyses conducted to assess robustness of the synthesized results.	Lines 192-195
Reporting bias assessment	14	Describe any methods used to assess risk of bias due to missing results in a synthesis (arising from reporting biases).	Lines 160-174
Certainty assessment	15	Describe any methods used to assess certainty (or confidence) in the body of evidence for an outcome.	Lines 201-207
**RESULTS**			
Study selection	16a	Describe the results of the search and selection process, from the number of records identified in the search to the number of studies included in the review, ideally using a flow diagram.	Lines 223-227 + Figure 1
	16b	Cite studies that might appear to meet the inclusion criteria, but which were excluded, and explain why they were excluded.	Lines 126-137
Study characteristics	17	Cite each included study and present its characteristics.	Lines 232-244 + Table 1
Risk of bias in studies	18	Present assessments of risk of bias for each included study.	Lines 423-436 + Supplementary Material - 3
Results of individual studies	19	For all outcomes, present, for each study: (a) summary statistics for each group (where appropriate) and (b) an effect estimate and its precision (e.g. confidence/credible interval), ideally using structured tables or plots.	Tables 2,3,4,5 + Figures 2,3,4,5,6
Results of syntheses	20a	For each synthesis, briefly summarise the characteristics and risk of bias among contributing studies.	Lines 232-244, 423-436
	20b	Present results of all statistical syntheses conducted. If meta-analysis was done, present for each the summary estimate and its precision (e.g. confidence/credible interval) and measures of statistical heterogeneity. If comparing groups, describe the direction of the effect.	Lines 344-373
	20c	Present results of all investigations of possible causes of heterogeneity among study results.	Lines 376-403
	20d	Present results of all sensitivity analyses conducted to assess the robustness of the synthesized results.	Lines 376-403
Reporting biases	21	Present assessments of risk of bias due to missing results (arising from reporting biases) for each synthesis assessed.	Lines 423-446
Certainty of evidence	22	Present assessments of certainty (or confidence) in the body of evidence for each outcome assessed.	Lines 435-446
**DISCUSSION**			
Discussion	23a	Provide a general interpretation of the results in the context of other evidence.	Lines 449-559
	23b	Discuss any limitations of the evidence included in the review.	Lines 562-568

Section and Topic	Item #	Checklist item	Location where item is reported
	23c	Discuss any limitations of the review processes used.	Lines 562-568
	23d	Discuss implications of the results for practice, policy, and future research.	Lines 571-580
**OTHER INFORMATION**			
Registration and protocol	24a	Provide registration information for the review, including register name and registration number, or state that the review was not registered.	Line 99
	24b	Indicate where the review protocol can be accessed, or state that a protocol was not prepared.	Line 99
	24c	Describe and explain any amendments to information provided at registration or in the protocol.	No amendments
Support	25	Describe sources of financial or non-financial support for the review, and the role of the funders or sponsors in the review.	Lines 612-613
Competing interests	26	Declare any competing interests of review authors.	Line 619
Availability of data, code and other materials	27	Report which of the following are publicly available and where they can be found: template data collection forms; data extracted from included studies; data used for all analyses; analytic code; any other materials used in the review.	Line 614

From: Page MJ, McKenzie JE, Bossuyt PM, Boutron I, Hoffmann TC, Mulrow CD, et al. The PRISMA 2020 statement: an updated guideline for reporting systematic reviews. BMJ 2021;372:n71. doi: 10.1136/bmj.n71. This work is licensed under CC BY 4.0. To view a copy of this license, visit https://creativecommons.org/licenses/by/4.0/

Table S3. Risk of Bias Assessment.

Study	Year	Design	Sample Size	Risk of Bias Assessment Domains	Overall Quality	Comments
**RANDOMIZED CONTROLLED TRIALS (Cochrane Risk of Bias Tool):**						
-	**Domain 1: Random Sequence Generation**	**Domain 2: Allocation Concealment**	**Domain 3: Blinding of Participants/Personnel**	**Domain 4: Blinding of Outcome Assessment**	**Domain 5: Incomplete Outcome Data**	**Domain 6: Selective Reporting**
**Louis et al.**	2023	RCT	207	⊕ Low risk - Computer-generated randomization	⊕ Low risk - Central allocation system	⊖ High risk[1] - Open-label design
**Wenzl et al.**	2015	RCT	52	⊕ Low risk - Computer randomization	⊕ Low risk - Sealed envelopes	⊕ Low risk[2] - Placebo-controlled double-blind
**Van Assche et al.**	2008	RCT	80	⊕ Low risk - Random number table	⊕ Low risk - Sealed envelopes	⊖ High risk[1] - Open-label withdrawal design
**Lémann et al.**	2005	RCT	83	⊕ Low risk - Computer-generated	⊕ Low risk - Central randomization	⊕ Low risk[2] - Double-blind placebo design
**Vilien et al.**	2004	RCT	29	⊕ Low risk - Random allocation stated	⊕ Low risk - Adequate concealment	⊖ High risk[1] - Open-label design
**Hawthorne et al.**	1992	RCT	67	◐ Unclear risk[10] - Limited methodological detail	◐ Unclear risk[10] - Allocation method unclear	⊕ Low risk[2] - Placebo-controlled design
**O'Donoghue et al.**	1978	RCT	51	◐ Unclear risk[10] - Randomization method	◐ Unclear risk[10] - Concealme	⊕ Low risk[2] - Double-blind

				not described	nt not described	placebo design
**Nyman et al.**	1985	RCT	42	◐ Unclear risk[10] - Random allocation stated only	◐ Unclear risk[10] - No concealment details	⊖ High risk[1] - Open-label design
**OBSERVATIONAL STUDIES (Newcastle-Ottawa Scale):**						
-	**Selection (4 stars max)**	**Comparability (2 stars max)**	**Outcome (3 stars max)**	**Comparability**	**Assessment of Outcome**	**Follow-up Length**
	**Representativeness**	**Selection of Controls**	**Ascertainment of Exposure**			
**Crepaldi et al.**	2023	Retrospective Cohort	274	★ Representative IBD population	★ Adequate control selection	★ Secure medical records
**Ranjan et al.**	2022	Retrospective Cohort	218	★ Representative multicenter cohort	★ Consecutive patient selection	★ Medical record validation
**Jorissen et al.**	2021	Retrospective Cohort	91	☆[16] Elderly-specific population (>60 years)	★ Adequate control group	★ Medical record review
**Cassinotti et al.**	2021	Prospective Cohort	57	★ Representative IBD patients	★ Prospective enrollment	★ Standardized exposure assessment
**Iborra et al.**	2019	Multicenter Observational	95	★ Multicenter representative sample	★ Consecutive enrollment	★ Registry data validation
**Moreno-Rincón et al.**	2015	Retrospective Cohort	102	★ UC-specific representative cohort	★ Consecutive patient selection	★ Medical record review
**Kennedy et al.**	2014	Retrospective Cohort	237	★ Large multicenter population	★ Systematic patient identification	★ Medical record validation

**Oussalah et al.**	2010	Retrospective Cohort	48	★ Combination therapy cohort	★ Well-defined control group	★ Medical record review
**Sokol et al.**	2010	Retrospective Cohort	47	★ Representative CD population	★ Consecutive enrollment	★ Medical record validation
**Angelucci et al.**	2010	Retrospective Cohort	41	☆[18] Abstract publication - insufficient detail	☆[18] Selection criteria unclear	★ Medical records mentioned
**Treton et al.**	2009	Prospective Cohort	66	★ Representative CD population	★ Prospective consecutive enrollment	★ Standardized data collection
**Cassinotti et al.**	2009	Retrospective Cohort	127	★ Large UC-specific cohort	★ Consecutive patient selection	★ Medical record validation
**Holtmann et al.**	2006	Retrospective Cohort	1176	★ Very large multicenter sample	★ Systematic case identification	★ Medical record review
**Fraser et al.**	2002	Retrospective Cohort	222	★ Large representative IBD cohort	★ Systematic patient identification	★ Medical record review

RoB Tool Explanations: *[1] Open-label design inevitable; [2] Placebo-controlled design; [3] Early termination for slow recruitment; [4] Early termination bias; [5] Not powered for non-inferiority; [6] Loss to follow-up >10%; [7] Unblinded outcome assessment; [8] High dropout rate; [9] Very small sample, underpowered; [10] Limited methodology reporting in historical studies; [11] Differential dropout rates; [12] Significant loss to follow-up; [13] Historical unblinded assessment; [14] Limited outcome reporting; [15] Small historical cohort with selection issues.* ***NOS Explanations:*** *[16] Elderly-specific population limits representativeness; [17] Limited comparability analysis; [18] Abstract publication with insufficient detail; [19] No comparability analysis reported; [20] Undefined clinical relapse assessment; [21] Insufficient follow-up detail; [22] Limited outcome assessment detail; [23] Heterogeneous follow-up across centers.* ***Legend:*** *⊕ Low risk of bias; ◐ Unclear risk of bias; ⊖ High risk of bias; ★ NOS criterion met (1 point); ☆ NOS criterion not met (0 points).*

Table S4. GRADE Evidence Assessment Framework.

Outcome	Risk of Bias	Inconsistency	Indirectness	Imprecision	Publication Bias	Other Considerations	Quality of Evidence	Summary of Findings
**PRIMARY OUTCOMES:**								
**Overall relapse incidence**	Serious limitations[−1]	Very serious inconsistency[−2]	No serious indirectness	No serious imprecision[3]	Undetected[4]	Large effect size[+1]	⊕⊕⊖⊖ **LOW**	32.5% (95% CI: 28.2-37.2%). Moderate evidence that 1 in 3 patients relapse after AZA withdrawal
**UC-specific relapse rates**	Serious limitations[−1]	Very serious inconsistency[−2]	No serious indirectness	Serious imprecision[−5]	Undetected[4]	Large effect size[+1]	⊕⊖⊖⊖ **VERY LOW**	41.3% (95% CI: 32.6-50.6%). Very limited confidence that UC patients have higher relapse rates
**CD-specific relapse rates**	Serious limitations[−1]	Very serious inconsistency[−2]	No serious indirectness	No serious imprecision[3]	Undetected[4]	None	⊕⊕⊖⊖ **LOW**	24.7% (95% CI: 19.8-30.3%). Low confidence that CD patients have lower relapse rates than UC
**UC vs CD comparison**	Serious limitations[−1]	Serious inconsistency[−6]	No serious indirectness	No serious imprecision[3]	Undetected[4]	Large effect size[+1]	⊕⊕⊖⊖ **LOW**	UC relapse risk significantly higher than CD (P=0.003). Low confidence in disease-specific difference
**Monotherapy**	Serious limitations[−1]	Very serious	No serious	No serious	Undetected[4]	Large effect size[+1]	⊕⊕⊖⊖ **LOW**	32.5% (95% CI: 27.8-37.5%). Low

**relapse rates**		inconsistency[−2]	indirectness	imprecision[3]				confidence in monotherapy withdrawal outcomes
**Combination therapy relapse rates**	Serious limitations[−1]	Serious inconsistency[−6]	No serious indirectness	Very serious imprecision[−7]	Undetected[4]	None	⊕⊖⊖⊖ **VERY LOW**	33.1% (95% CI: 26.2-40.7%). Very limited confidence in combination therapy outcomes
**SECONDARY OUTCOMES:**								
**Time to relapse**	Serious limitations[−1]	Very serious inconsistency[−2]	No serious indirectness	Very serious imprecision[−7]	Undetected[4]	None	⊕⊖⊖⊖ **VERY LOW**	Median 12-21 months. Very limited confidence in time-to-relapse estimates
**CRP as relapse predictor**	Serious limitations[−1]	Serious inconsistency[−6]	No serious indirectness	Serious imprecision[−5]	Undetected[4]	Consistent direction[+2]	⊕⊕⊖⊖ **LOW**	Elevated CRP (>5-20 mg/L) associated with increased relapse risk. Low confidence in predictive value
**Fecal calprotectin as predictor**	Serious limitations[−1]	No serious inconsistency	No serious indirectness	Very serious imprecision[−7]	Undetected[4]	Strong association[+3]	⊕⊖⊖⊖ **VERY LOW**	FC >50-300 μg/g associated with relapse. Very limited confidence due to few studies
**Mucosal healing as predictor**	Serious limitations[−1]	Serious inconsistency[−6]	No serious indirectness	Very serious imprecision[−7]	Undetected[4]	Strong protective effect[+3]	⊕⊖⊖⊖ **VERY LOW**	Mucosal healing protective against relapse. Very limited

								confidence in effect size
**AZA duration predictor**	Serious limitations[−1]	Serious inconsistency[−6]	No serious indirectness	Serious imprecision[−5]	Undetected[4]	Dose-response relationship[+4]	⊕⊕⊖⊖ **LOW**	Shorter AZA duration (<4 years) increases relapse risk. Low confidence in optimal duration
**Age as predictor**	Serious limitations[−1]	Very serious inconsistency[−2]	No serious indirectness	Very serious imprecision[−7]	Undetected[4]	None	⊕⊖⊖⊖ **VERY LOW**	Conflicting age effects across studies. Very limited confidence in age-based recommendations
**Gender as predictor**	Serious limitations[−1]	Very serious inconsistency[−2]	No serious indirectness	Very serious imprecision[−7]	Undetected[4]	None	⊕⊖⊖⊖ **VERY LOW**	Inconsistent gender effects. Very limited confidence in gender-based risk stratification
**POST-RELAPSE MANAGEMENT OUTCOMES:**								
**AZA re-treatment success**	Very serious limitations[−8]	No serious inconsistency	No serious indirectness	Very serious imprecision[−7]	Undetected[4]	Large effect size[+1]	⊕⊖⊖⊖ **VERY LOW**	High re-treatment success (60-100%). Very limited confidence due to small numbers and selection bias
**Biologic escalation rates**	Serious limitations[−1]	Serious inconsistency[−6]	No serious indirectness	Very serious imprecision[−7]	Undetected[4]	None	⊕⊖⊖⊖ **VERY LOW**	Variable escalation to biologics (10-30%). Very limited confidence in escalation patterns

**Surgery after relapse**	Serious limitations[−1]	Serious inconsistency[−6]	No serious indirectness	Very serious imprecision[−7]	Undetected[4]	None	⊕⊖⊖⊖ **VERY LOW**	Low surgery rates (2-15%). Very limited confidence in surgical risk estimates

***Abbreviations**: AZA, azathioprine; CI, confidence interval; CD, Crohn's disease; CRP, C-reactive protein; FC, fecal calprotectin; GRADE, Grading of Recommendations Assessment, Development and Evaluation; RCT, randomized controlled trial; UC, ulcerative colitis.*
